# Co-reaction accelerators for electrochemiluminescence: from fundamental mechanisms to advanced biosensing

**DOI:** 10.1039/d6sc05426a

**Published:** 2026-08-21

**Authors:** Jingxian Li, Dan Yang, Ren Cai, Weihong Tan

**Affiliations:** a Hunan Key Laboratory of Molecular Epidemiology, School of Public Health, Hunan Normal University Changsha Hunan 410013 China; b Molecular Science and Biomedicine Laboratory, State Key Laboratory of Chemo and Biosensing, College of Material Science and Engineering, College of Chemistry and Chemical Engineering, Hunan University Changsha Hunan 410082 China cairen@hnu.edu.cn; c The Cancer Hospital of the University of Chinese Academy of Sciences (Zhejiang Cancer Hospital), Hangzhou Institute of Medicine (HIM), Chinese Academy of Sciences Hangzhou Zhejiang 310022 China; d Institute of Molecular Medicine, Renji Hospital, Shanghai Jiao Tong University School of Medicine, College of Chemistry and Chemical Engineering, Shanghai Jiao Tong University Shanghai 200240 China; e Department of Chemical Engineering, The University of Western Australia Perth Western Australia 6009 Australia dan.yang@uwa.edu.au

## Abstract

Electrochemiluminescence (ECL) is a sensitive analytical technique, which converts electrochemical energy into electromagnetic radiation. With its high sensitivity, low background noise, and excellent controllability, ECL demonstrates significant application potential in fields such as bioanalysis, environmental monitoring, and food safety. However, traditional co-reactant-based ECL systems often suffer from low reaction efficiency and require relatively high concentrations of co-reactants to achieve satisfactory luminescence performance. The introduction of co-reaction accelerators (CRAs) offers an innovative strategy for constructing efficient and stable ECL systems by promoting the generation and transformation of reactive co-reactant intermediates. This review provides a comprehensive overview of the latest advances in CRAs. First, based on material composition, CRAs are categorized into three major categories: inorganic CRAs, bio-organic CRAs, and multifunctional CRAs. The fundamental principles of ECL and the underlying mechanisms of CRA-mediated signal enhancement are then discussed. We also summarize recent achievements of CRA-based ECL sensing platforms in detecting disease biomarkers and environmental pollutants, as well as in food safety applications. Finally, we discuss current challenges in this field and outline future development directions, aiming to provide valuable insights for the rational design of next-generation high-performance ECL analytical technologies.

## Abstract 2

This review categorizes current CRAs into inorganic, bio-organic and multifunctional types, clarifies their ECL signal enhancement mechanisms, and pinpoints key existing challenges.

## Graphical abstract

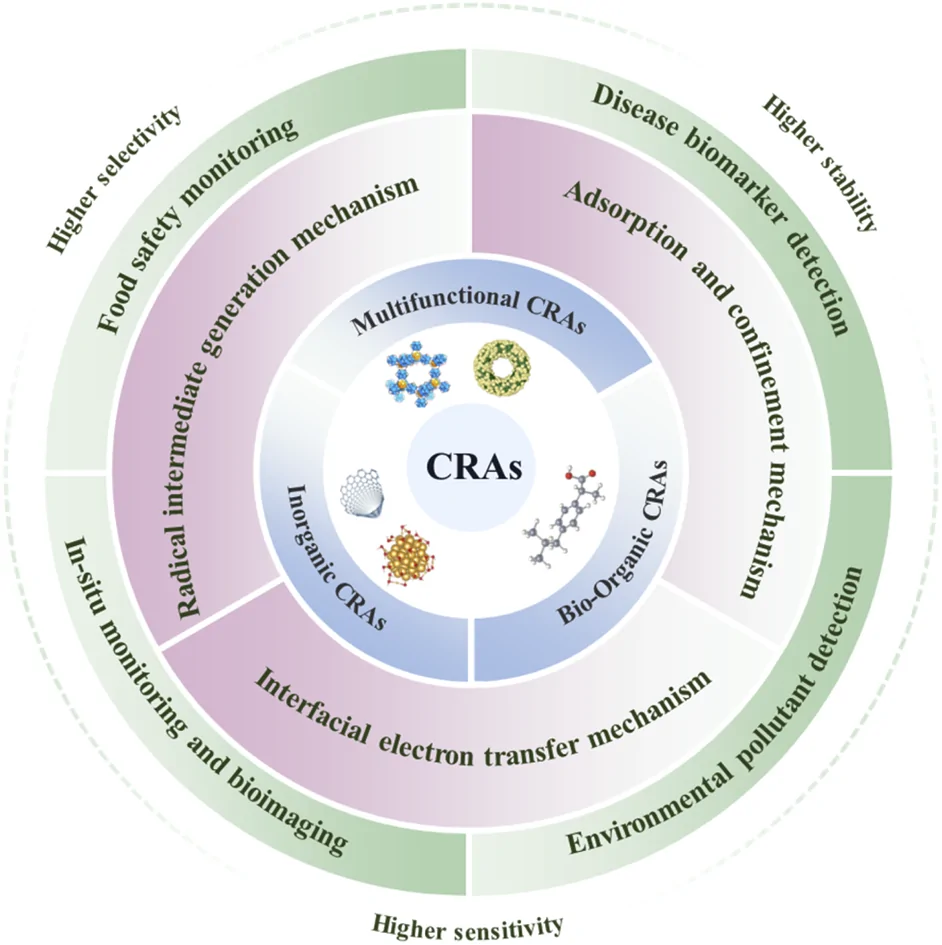

## 1. Introduction

Electrochemiluminescence (ECL), also termed electrogenerated chemiluminescence, stands as an interdisciplinary analytical technology that couples electrochemical triggering with chemiluminescent radiation, integrating their core advantages, including near-zero background signal interference, broad linear detection range, mild and controllable reaction conditions, simplified analytical equipment configuration, and outstanding adaptability to complex biological sample matrices.^1–5^ Since its initial discovery and systematic mechanistic exploration, ECL has gradually developed into one of the most competitive analytical tools in contemporary bioanalysis, with extensive applications spanning clinical disease diagnosis, early tumor biomarker screening, trace nucleic acid detection, environmental pollutant monitoring, and food safety testing.^6–9^ Unlike annihilation ECL, which relies on redox interactions between oxidized and reduced luminophore radicals generated at extremely high or low potentials, co-reactant-mediated ECL has become the mainstream system in practical bioanalytical scenarios, as it enables efficient luminescent emission under moderate aqueous potential conditions while avoiding the limitations associated with severe electrode polarization and harsh electrochemical operating potentials.^10–12^

Although conventional co-reactant ECL systems (such as Ru(bpy)_3_^2+^–tripropylamine (TPrA), luminol–H_2_O_2_, and semiconductor quantum dot–K_2_S_2_O_8_) have been widely applied in routine analysis, critical limitations still restrict their performance in high-sensitivity detection of ultra-trace biological targets. The inherent low efficiency of electrochemical conversion from co-reactants to highly active radical intermediates, sluggish heterogeneous electron transfer dynamics at the electrode interface, and serious radical quenching effects jointly lead to limited luminescence quantum yield and unsatisfactory detection sensitivity, which hinder further application of traditional ECL systems in early disease diagnosis and ultra-trace target quantitative analysis.^13^ In response to these technical bottlenecks, co-reaction accelerators (CRAs) have emerged as novel functional materials and molecular regulators, providing a highly efficient signal amplification strategy without altering the intrinsic structure of luminophores or replacing conventional co-reactants.

**Scheme 1:**
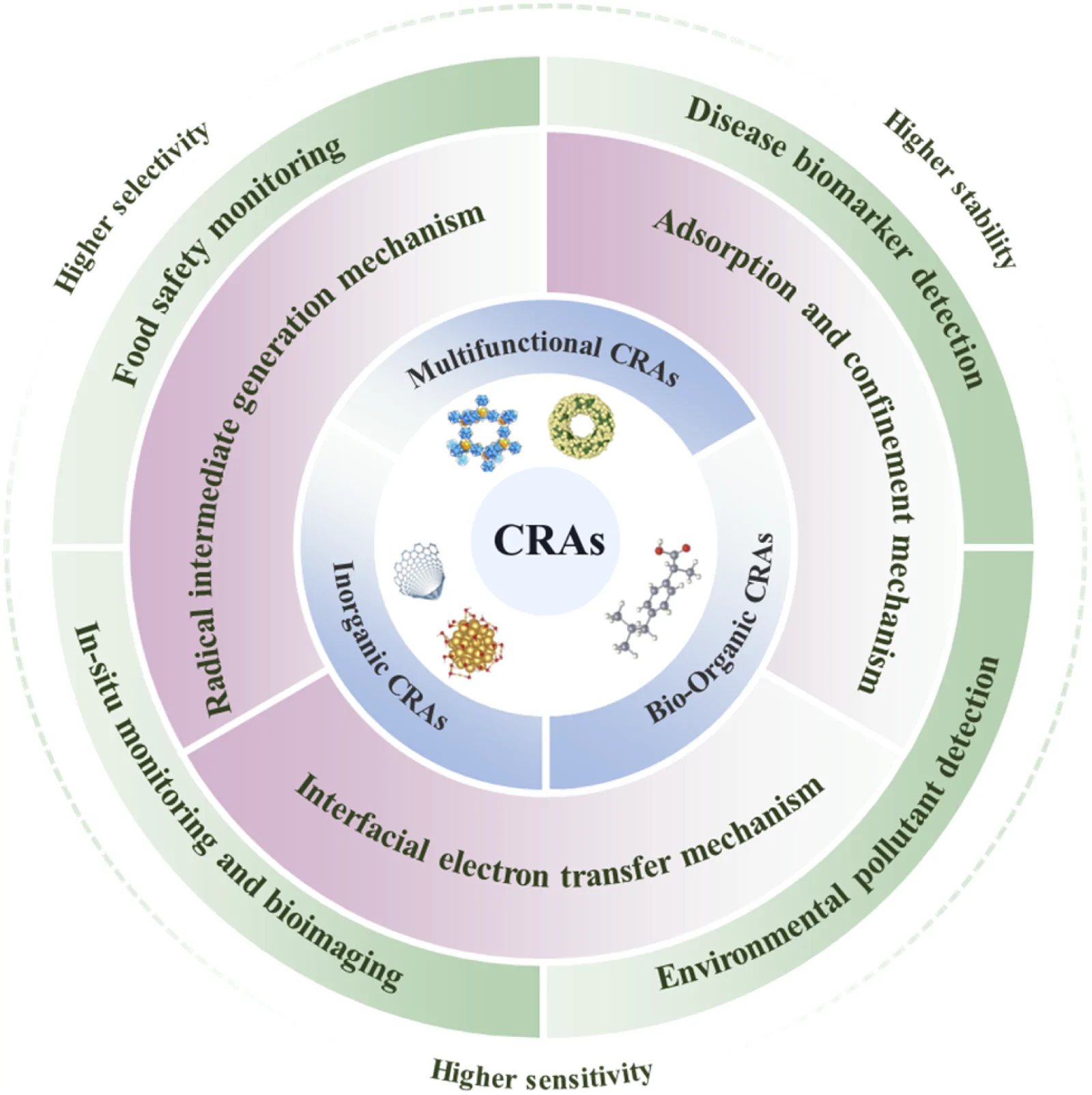
A schematic overview of the CRAs classification, ECL enhancement mechanisms, and ECL biosensing applications.

CRAs can be defined as trace-dose functional substances (covering nanomaterials, small organic molecules, biomimetic catalysts, and multi-component composites) that selectively catalyze the electrochemical redox decomposition of co-reactants, boost the rapid and large-scale generation of active radical intermediates, accelerate interfacial electron transfer efficiency, and inhibit non-radiative radical quenching, thereby indirectly and significantly enhancing ECL emission intensity without directly participating in the excitation process of luminophores.^14–19^ In contrast to conventional co-reactants or luminescent probe modification strategies, CRAs feature superior catalytic efficiency, ultra-low dosage demand, excellent compatibility with diverse ECL systems, and flexible structural designability, making them a research focus in the ECL field over the past decade. This review systematically categorizes cutting-edge CRA materials and corresponding rational design strategies, expounds the fundamental catalytic mechanisms of advanced CRAs, summarizes their applications in high-performance ECL bioanalysis, discusses feasible performance optimization approaches, and forecasts future development trends and unresolved challenges. We aim to provide a comprehensive theoretical reference and technical guidance for researchers engaged in ECL sensing, bioanalytical chemistry, and functional nanomaterial development (Scheme 1).

## 2. Classification and design strategies of advanced CRAs

According to material composition and structural characteristics, advanced CRAs are mainly divided into three major categories: inorganic material-based CRAs, organic and biomolecule-based CRAs, and composite multifunctional CRAs. Each category has unique catalytic properties, application scenarios, and targeted design strategies to further optimize the ECL amplification performance.

### 2.1 Inorganic CRAs

Inorganic CRAs dominate current research due to their high electrocatalytic activity, excellent stability, and adjustable structure, and are further divided into noble metal nanomaterials, single-atom nanomaterials, transition metal nanomaterials, and carbon-based nanomaterials.

#### 2.1.1 Noble metal nanomaterials

Noble metal nanomaterials (including Au, Ag, Pt, Pd, and their binary/multinary alloys) are the earliest and most extensively studied CRAs, characterized by ultra-high electrical conductivity, exceptional electrocatalytic activity, and favorable biocompatibility. Their catalytic performance is highly dependent on morphological and dimensional regulation: zero-dimensional nanoparticles, one-dimensional nanowires, two-dimensional nanosheets, and three-dimensional nanoflower structures exhibit distinct active site densities and electron transfer efficiencies.

**Fig. 1:**
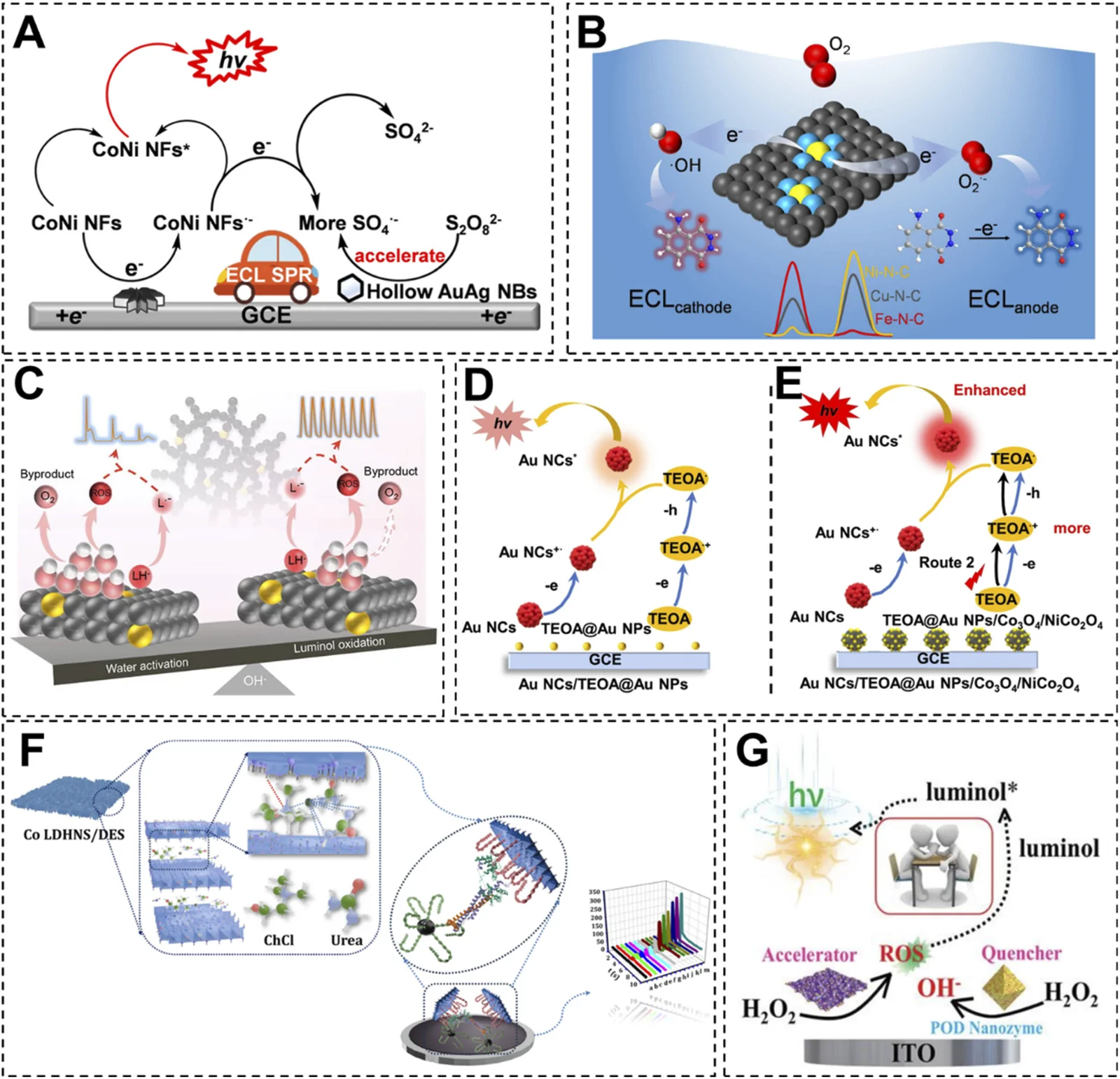
Inorganic CRAs utilized in ECL. (A) Hollow AuAg nanoboxes as CRA for the CoNi NFs/S_2_O_8_^2−^ system. Reproduced with permission from ref. 20. Copyright (2024) American Chemical Society. (B) M–N–C SACs as CRA to promote the ECL of luminol. Reproduced with permission from ref. 23. Copyright (2024) American Chemical Society. (C) NiIr single-atom alloy aerogels as CRA to improve the water activation activity and durability. Reproduced with permission from ref. 24. Copyright (2024) Elsevier. (D and E) Au NPs/Co_3_O_4_/NiCo_2_O_4_ as CRA to improve the ECL efficiency of gold nanoclusters. Reproduced with permission from ref. 29. Copyright (2025) Elsevier. (F) Co LDH NSs/DES as CRA to enhance the ECL of luminol. Reproduced with permission from ref. 30. Copyright (2026) Elsevier. (G) ZIF-67@MXene nanosheet as CRA to enhance the ECL of luminol. Reproduced with permission from ref. 43. Copyright (2024) Wiley-VCH.

For instance, novel hollow AuAg nanoboxes were prepared and employed as a CRA to enhance the ECL performance of CoNi nanofibers (NFs) in a cathodic K_2_S_2_O_8_ system by catalyzing the generation of additional sulfate radicals (Fig. 1A).^20^ Compared with individual Au and Ag nanoparticles, the hollow AuAg nanoboxes exhibited superior ECL enhancement, highlighting the synergistic effect of alloying. In a separate study, PdPt nanosheets were developed to promote the ECL of SnS_2_ nanosheets, demonstrating that PdPt nanosheets facilitate sulfate radical generation and thereby amplify the ECL signal.^21^

Noble metal CRAs rely on their ultra-high conductivity and surface plasmon effects to promote electron transfer. Alloying further tunes the d-band center, which optimizes the adsorption energy of active sites. Their main advantages are exceptionally high catalytic activity, strong morphological controllability, and good biocompatibility. However, they suffer from high cost, limited reserves, and potential long-term biotoxicity. These drawbacks restrict their large-scale application in ECL.

#### 2.1.2 Single-atom nanomaterials

Single-atom nanomaterials, characterized by unprecedented 100% atomic utilization, atomically precise coordination environments, and tunable electronic structures, have redefined the paradigm of co-reactant design in ECL systems.^22^ Unlike conventional molecular or nanoscale promoters, single-atom catalysts offer unparalleled control over reactive oxygen species (ROS) generation pathways, thereby addressing long-standing limitations in signal specificity, triggering potential, and operational stability. This emerging class of single-atom materials has rapidly ascended as a cornerstone of next-generation ECL sensor development, particularly in the context of complex biological matrices where multiplexed detection is paramount.

The advent of potential-resolved ECL platforms with dual-emission signatures represents a transformative leap toward unambiguous analyte discrimination in heterogeneous samples. Yet, their practical deployments have been persistently hampered by cross-talk between disparate luminophores and mismatched co-reactant kinetics. To circumvent this fundamental challenge, Zhou *et al.* designed a novel strategy that leverages endogenous dissolved oxygen as the sole co-reactant to enable by meticulously engineered M–N–C single-atom catalysts (SACs).^23^ Through the synergistic *in situ* spectroscopic interrogation and density functional theory calculations, they revealed that the central metal center governs the selective activation of O_2_ into two distinct ROS: hydroxyl radicals (˙OH) and superoxide anions (O_2_˙^−^), which are spatially and temporally segregated to trigger cathodic and anodic luminol emissions, respectively (Fig. 1B). Crucially, the Cu–N–C SAC with the optimized oxygen affinity uniquely balances the dual-generation kinetics of these radicals, thereby achieving intrinsic potential-resolved dual-signal output without external additives.

Conversely, the anodic oxygen evolution reaction (OER), long regarded as a detrimental side process in aqueous ECL systems, has recently been reinterpreted not as a nuisance, but as a tunable resource. Ling *et al.* demonstrated that the ROS was generated during water oxidation and can be harnessed to modulate ECL intensity.^24^ By employing a NiIr single-atom alloy aerogel with exceptional OER activity, they established a direct mechanistic link among hydroxide ion concentration, water oxidation rate, and luminol emission efficiency (Fig. 1C). Remarkably, lowering the concentration of OH^−^ could suppresses O_2_ accumulation at the electrode surface, mitigate signal quenching, and simultaneously enhance reproducibility. This counterintuitive approach transforms a classical parasitic reaction into a dynamic control knob, offering a paradigm shift in ECL system design: their kinetics can be engineered to serve analytical purposes rather than suppressing side reactions.

In summary, single-atom CRAs rely on precise electronic regulation and coordination engineering of central metal sites. They solve two important problems in traditional ECL systems. One is the uncontrollable generation path of reactive oxygen species. The other is severe interference from side reactions. They also achieve multiple key performance breakthroughs. These breakthroughs include low triggering potential, dual-signal resolution and excellent long-term stability. Such materials provide brand-new design ideas for building new ECL sensing platforms. These platforms feature high sensitivity and high specificity. They also open a new research direction for the cross-integration of single-atom catalysis and analytical chemistry. They show extremely high application potential in scenarios, like trace biomarker detection and complex environmental sample analysis.

#### 2.1.3 Transition metal nanomaterials

Transition metal compounds, including oxides, hydroxides, sulfides, phosphides, and layered double hydroxides (LDHs), have emerged as ideal substitutes for noble metal CRAs owing to their rich variable valence states, tunable electronic structures, and low cost. Representative materials, such as Co_3_O_4_, NiFe-LDH, MoS_2_, NiCoP, and CuS, exhibit excellent co-reactant catalytic activity, particularly in luminol and semiconductor quantum dot ECL systems.^25–30^ Elemental doping (*e.g.*, N, P, Fe) and heterostructure construction further expose more catalytic active sites, optimizing electronic conductivity, and enhancing long-term stability.^31^

A notable example is the work by Zhang *et al.*, who addressed the inherently low ECL efficiency of gold nanoclusters (Au NCs) by introducing Au NPs/Co_3_O_4_/NiCo_2_O_4_ as a novel CRA.^32^ This ternary catalyst efficiently oxidizes triethanolamine (TEOA) to generate a large amount of the reducing intermediate radical TEOA˙^+^, thereby enabling a significantly enhanced ECL response from Au NCs (Fig. 1D and E). For anodic luminol ECL systems, performance is often limited by insufficient generation of reactive oxygen species (ROS), which serve as essential co-reactants. Conventional co-reactants frequently suffer from biological toxicity or instability, while dissolved oxygen exhibits negligible reactivity due to its low solubility. To overcome this limitation, Khoshfetrat *et al.* designed a deep eutectic solvent (DES)-assisted cobalt layered double hydroxide nanosheet (Co LDH NSs/DES) system that efficiently catalyzes the oxygen evolution reaction (OER) to achieve *in situ* ROS production (Fig. 1F).^33^ The DES expands the interlayer spacing of Co-LDH NSs, providing abundant catalytic sites and facilitating electrolyte penetration, mass transfer, and electron transfer, which collectively accelerate OER kinetics and stabilize ROS output. This OER-mediated ECL (OER-ECL) strategy ensures continuous ROS generation in the vicinity of luminol radicals, significantly amplifying the ECL signal. Notably, water has been demonstrated for the first time to serve as an innovative co-reactant in the luminol ECL system.^34^ In this configuration, cobalt–iron layered double hydroxide (CoIr LDH) serves as a state-of-the-art CRAs, which can efficiently catalyze the oxidation of water to generate reactive oxygen species, thus enabling significantly amplified ECL emission for sensitive biosensing applications.

Transition metal CRAs catalyze reactions through rich redox cycling of variable valence states (*e.g.*, Co^2+^/Co^3+^, Cu^+^/Cu^2+^). This enables efficient activation of co-reactants and *in situ* ROS generation. Their advantages include low cost, abundant reserves, and flexible tunability of electronic structures *via* doping and heterostructure construction. However, their intrinsic conductivity is generally lower than that of noble metals. In addition, some sulfides and phosphides suffer from structural degradation during long-term electrochemical cycling. The structure–activity relationships of these materials also remain to be systematically clarified.

#### 2.1.4 Carbon-based nanomaterials

Carbon-based CRAs, including graphene, carbon nanotubes (CNTs), MXene, graphitic carbon nitride (g-C_3_N_4_), and carbon dots (CDs), feature ultra-high specific surface area, excellent electrical conductivity, good biocompatibility, and facile surface functionalization.^35–43^ They serve dual roles as rapid electron transfer carriers and co-reactant adsorption substrates, realizing both catalytic amplification and matrix support.^44,45^ Heteroatom doping (N, S, P, B) introduces lattice defect sites and modulates surface charge distribution, significantly improving electrocatalytic activity toward co-reactants. For example, N-doped graphene and Ti_3_C_2_ MXene nanosheets accelerate interfacial electron transfer and enrich co-reactant molecules, achieving remarkable ECL signal amplification.^46^

Beyond two-dimensional carbon materials, porous carbon composites have also shown great promise. Wang *et al.* designed ZIF-67@MXene nanosheet composites featuring highly conductive in-plane structures and confined, stable pores/channels as efficient CRAs, which generated abundant hydroxyl radicals (˙OH) and singlet oxygen (^1^O_2_) in a luminol–H_2_O_2_-based ECL system, leading to an 830% enhancement in luminol ECL efficiency (Fig. 1G).^47^

Carbon-based CRAs function through their high specific surface areas, which provide abundant adsorption sites. Heteroatom doping introduces defect states that optimize charge distribution. Their excellent conductivity also accelerates interfacial electron transfer. Key advantages include low cost, large surface area, easy surface functionalization, and good biocompatibility. However, their intrinsic catalytic activity is generally weaker than that of metal-based CRAs. Some materials, such as MXene, also suffer from oxidative instability in oxygen- or water-containing environments. Composite strategies are needed to address these limitations.

### 2.2 Bio-organic CRAs

Organic and biomolecule-based CRAs are characterized by good water solubility, easy structural modification, and high biological specificity, suitable for biocompatible and portable ECL sensing platforms.

#### 2.2.1 Organic molecule CRAs

Organic molecule CRAs, including metal–organic frameworks (MOFs) and organic polymer materials, promote co-reactant activation through hydrogen bonding, electron donation, and radical stabilization.

**Fig. 2:**
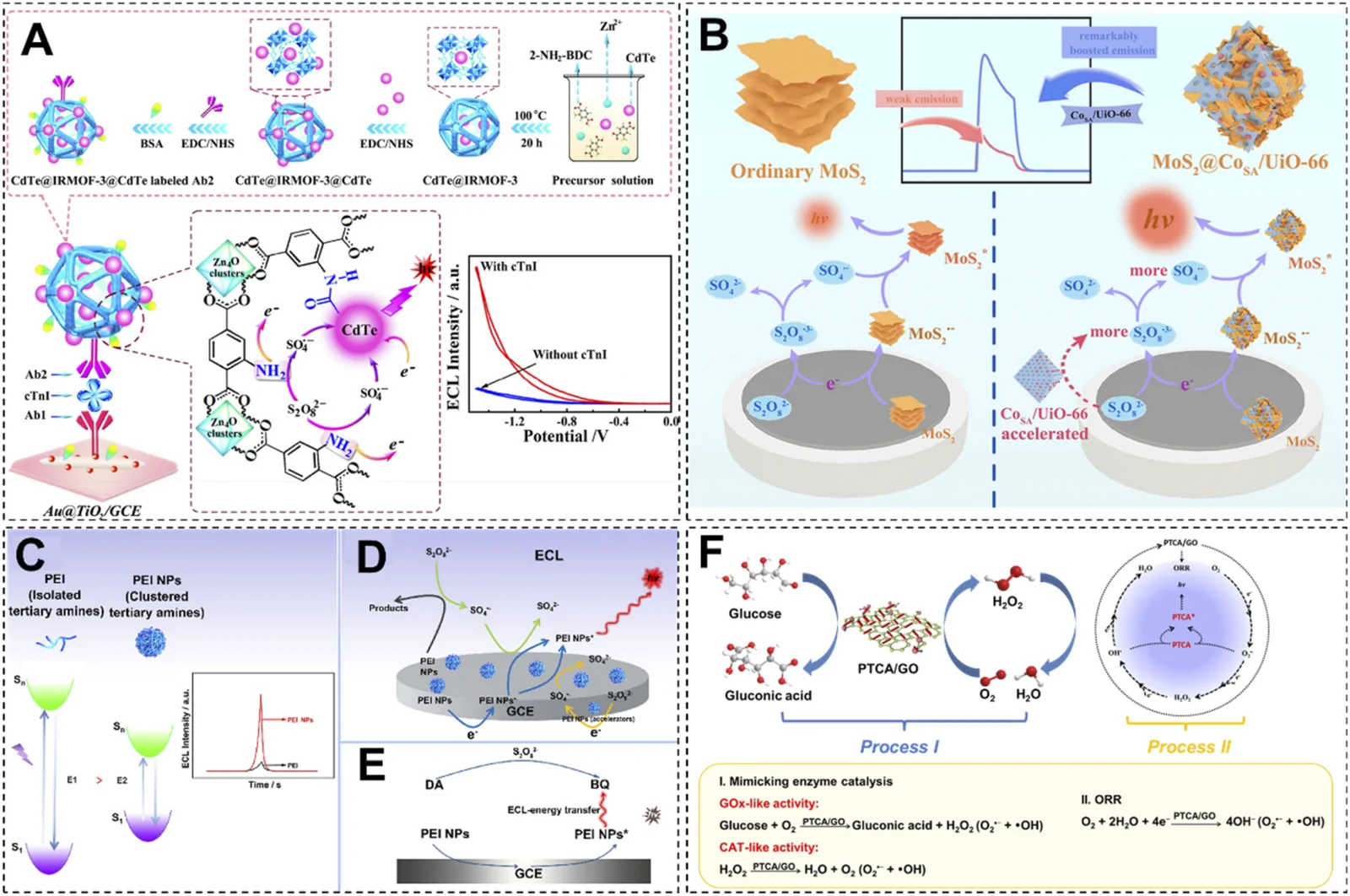
Organic molecule CRAs utilized in ECL. (A) IRMOF-3 as CRA to enhance the ECL emission of CdTe quantum dots. Reproduced with permission from ref. 16. Copyright (2018) American Chemical Society. (B) UiO-66 as CRA to boost the ECL emission of MoS_2_. Reproduced with permission from ref. 47. Copyright (2026) Elsevier. (C–E) PEI nanoparticles as CRA to enhance the ECL intensity of pure PEI. Reproduced with permission from ref. 50. Copyright (2022) American Chemical Society. (F) PTCA/GO nanozyme as CRA in the PTCA–glucose ECL system. Reproduced with permission from ref. 52. Copyright (2025) American Chemical Society.

MOFs, in particular, have attracted considerable attention owing to their large surface area, abundant active sites, and excellent water stability. For instance, Yang *et al.* employed reticular metal–organic framework 3 (IRMOF-3), constructed from 2-aminoterephthalic acid (2-NH_2_-BDC) as the organic ligand, as a co-reactant accelerator to enhance the ECL emission of CdTe quantum dots. This strategy established a new mechanism for significantly boosting ECL intensity: IRMOF-3 not only enables high loading of CdTe quantum dots *via* encapsulation and internal/external decoration but also serves as a novel co-reactant accelerator that promotes the conversion of S_2_O_8_^2−^ into sulfate radical anions (SO_4_˙^−^), thereby further amplifying the ECL signal of CdTe (Fig. 2A).^16^

Beyond conventional MOFs, SACs also hold significant potential for enhancing ECL efficiency.^22,48,49^ However, SACs reported in the ECL field typically rely on carbon–nitrogen-based substrates and require high-temperature pyrolysis (>800 °C), which limits active site density due to anchoring constraints and thermal degradation. To overcome these limitations, Zhao *et al.* developed a zirconium-based MOF, UiO-66, as a support to sequentially anchor cobalt single atoms (CoSA) and the ECL emitter MoS_2_ under mild conditions (<200 °C). The resulting CoSA/UiO-66 not only optimizes the electronic structure of MoS_2_ and effectively promotes the formation of Co(iv)=O but also establishes Mo–O–Zr bimetallic sites at the UiO-66/MoS_2_ interface that facilitate carrier migration within MoS_2_, efficiently catalyzing the reduction of S_2_O_8_^2−^. Accordingly, under step-pulse electrochemical excitation, the ECL emission of MoS_2_ at −1.6 V is significantly enhanced (Fig. 2B).^50^

Organic molecule CRAs, especially MOFs, function through confined catalysis in ligand–metal coordination environments. Hydrogen bonding and electron-donor effects facilitate co-reactant activation. SACs achieve precise catalysis through isolated metal active sites. Their advantages include highly designable structures, good water stability, low cost, and easy device integration. However, their intrinsic conductivity is poor and requires carbon-based composites to compensate. High-temperature synthesis can also damage active sites. In addition, the structural stability of MOFs under strong acid or alkaline conditions still needs improvement.

#### 2.2.2 Biomacromolecule and nanozyme CRAs

Biomacromolecule CRAs (*e.g.*, enzymes, peptides, DNA aptamers) and nanozyme CRAs (enzyme-mimetic inorganic nanomaterials) integrate specific biological recognition with ECL amplification in a single platform. Natural enzymes such as horseradish peroxidase (HRP) and glucose oxidase (GOx) catalyze co-reactant generation with high specificity but suffer from poor stability. Nanozymes overcome this limitation by offering high catalytic activity and robust stability under complex conditions.^51,52^

A representative example is the work by Zhang *et al.*, who synthesized polyethylenimine nanoparticles (PEI NPs) with cluster-based luminescence *via* a one-step hydrothermal method using PEI and formaldehyde (HCHO) as precursors (Fig. 2C).^53^ PEI NPs are non-conjugated polymer (NCP) nanoparticles featuring tertiary amine clusters and serve as ECL emitters. Systematic photochemical and electrochemical investigations revealed that the tertiary amines emit *via* a cluster-dominated mechanism *via* “through-space conjugation,” with their ECL performance (emission at 631 nm) further confirmed through an initiated reduction–oxidation process. Notably, when persulfate was employed as the co-reactant, PEI NPs functioned simultaneously as the luminophore and co-reactant accelerator, enhancing the ECL intensity to eight times that of pure PEI (Fig. 2D). On this basis, sensitive detection of dopamine was also achieved (Fig. 2E).

Despite these advances, most nanozymes reported in ECL systems are used solely as CRAs, with their intrinsic luminophore capability remaining underexploited.^54^ To address this gap, a PTCA/GO nanozyme was constructed with dual-enzyme mimetic activities, which functions not only as an ECL luminophore but also as an efficient co-reactant accelerator in the PTCA–glucose ECL system (Fig. 2F).^55^ This nanozyme exhibits both catalase-like (CAT-like) and glucose oxidase-like (GOx-like) activities. In the catalytic cascade, glucose is first oxidized to H_2_O_2_, which is subsequently decomposed into O_2_ and H_2_O, generating substantial reactive oxygen species (ROS). Concurrently, the four-electron-dominated oxygen reduction reaction (ORR) on PTCA/GO produces additional ROS (˙OH and O_2_˙^−^), both of which were confirmed to participate in the ECL emission of the PTCA–glucose system. This dual-function design broadens the application scope of nanozymes in ECL beyond their conventional role as co-reactant accelerators. These advances underscore a paradigm shift in which nanozymes evolve from single-function co-reactant accelerators into multifunctional platforms that unify biorecognition with ECL signal amplification, opening new avenues for high-performance sensing applications.

Biomacromolecule and nanozyme CRAs work by mimicking natural enzyme cascades (*e.g.*, CAT/GOx dual activity) or exploiting cluster luminescence for integrated “emission–catalysis” functions. Their advantages include excellent biocompatibility, high recognition specificity, and far superior stability of nanozymes over natural enzymes. However, natural enzymes suffer from poor stability. Nanozymes generally show lower activity than natural enzymes, and their batch-to-batch consistency needs improvement. Moreover, most nanozymes are used solely as CRAs in ECL systems. Their luminescence potential remains largely untapped.

### 2.3 Multifunctional CRAs

**Fig. 3:**
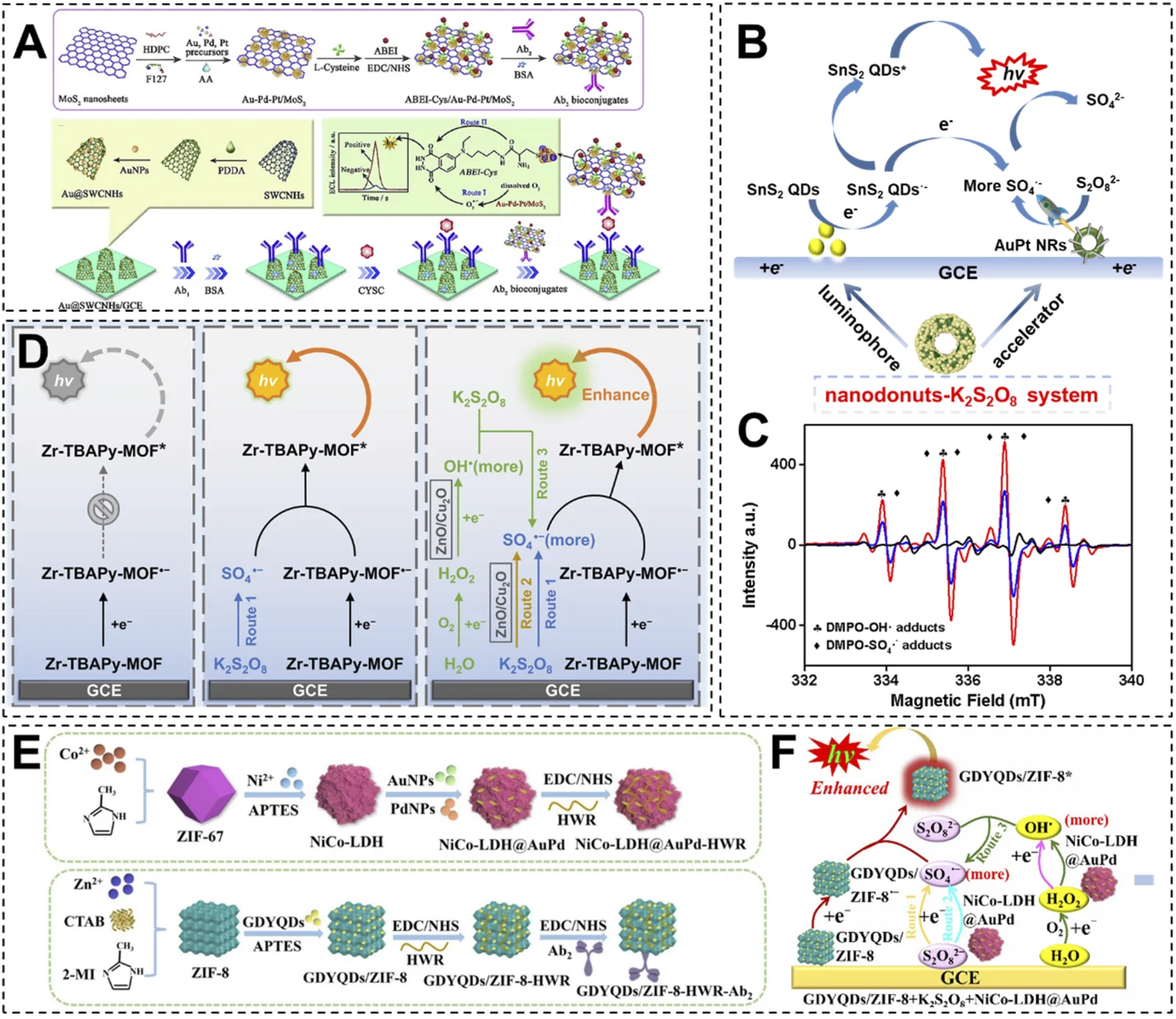
Multifunctional CRAs utilized in ECL. (A) Au–Pd–Pt/MoS_2_ as CRA for the ABEI/O_2_ ECL system. Reproduced with permission from ref. 53. Copyright (2020) Elsevier. (B and C) AuPt NRs as CRA in the SnS_2_ QD/K_2_S_2_O_8_ system. Reproduced with permission from ref. 54. Copyright (2023) American Chemical Society. (D) Au@ZnO/Cu_2_O as CRA to enhance the ECL intensity of AIE-LMOF. Reproduced with permission from ref. 56. Copyright (2024) Elsevier. (E and F) NiCo-LDH@AuPd as CRA to amplify the ECL signal of GDYQDs/ZIF-8. Reproduced with permission from ref. 57. Copyright (2025) American Chemical Society.

Multifunctional CRAs integrate the advantages of different materials, overcoming the limitations of single-component CRAs and achieving synergistic catalytic amplification. Typical examples include noble metal/transition metal compound composites, carbon-based nanomaterial/MOF composites, and biomolecule–inorganic hybrid CRAs. Li *et al.* developed an ultrasensitive ECL immunosensor for cystatin C (CysC) by combining a co-reactant accelerator with a self-enhanced ECL luminophore. Specifically, trimetallic Au–Pd–Pt nanoflower-functionalized MoS_2_ nanosheets (Au–Pd–Pt/MoS_2_) served as an efficient synergistic accelerator for the ABEI/O_2_ ECL system (Fig. 3A).^56^ In contrast, an integrated design was adopted by constructing a SnS_2_ quantum dot (QD)/Cys–AuPt hetero-nanoring (NR), which functions simultaneously as a highly efficient co-reactant accelerator and a label-free ECL aptasensor luminophore (Fig. 3B).^57^ In this heterostructure, SnS_2_ QDs are decorated on the AuPt NR surface, promoting the generation of additional co-reactive intermediates (SO_4_˙^−^) in the SnS_2_ QD/K_2_S_2_O_8_ system (Fig. 3C), thereby dramatically enhancing ECL performance.

Beyond noble metal composites, MOF-based CRAs have also demonstrated remarkable synergistic effects.^58^ Li *et al.* employed an aggregation-induced emission luminogen (AIEgen), 1,3,6,8-tetrakis(4-carboxyphenyl)pyrene (H_4_TBAPy), as the ligand and Zr_6_ clusters as nodes to synthesize a zirconium-based MOF (Zr-TBAPy-MOF). The resulting AIE-active luminescent MOF (AIE-LMOF) exhibited excellent ECL performance. Meanwhile, an Au@ZnO/Cu_2_O nanocomposite was designed as a CRA, in which the reversible Cu^+^/Cu^2+^ redox conversion in Cu_2_O significantly facilitates SO_4_˙^−^ generation from S_2_O_8_^2−^, while ZnO with superior electrochemical properties further boosts the overall catalytic activity (Fig. 3D).^59^ In a more recent study, Jia *et al.* employed graphdiyne quantum dot (GDYQD)-decorated zeolitic imidazolate framework-8 (GDYQDs/ZIF-8) as the ECL luminophore, paired with ZIF-67-derived NiCo layered double hydroxide (NiCo-LDH) supported with AuPd nanoparticles (NiCo-LDH@AuPd) as the CRA (Fig. 3E and F).^60^ The ZIF-67-derived NiCo-LDH combines the merits of both ZIF-67 and LDH to form a unique 3D nanocage architecture with hierarchical nanoarrays resembling mussels, which are formed by interconnected ultrathin nanosheets, thereby improving charge transfer efficiency. The synergistic interaction between NiCo-LDH and AuPd NPs in NiCo-LDH@AuPd further accelerates the reduction of K_2_S_2_O_8_ to generate more SO_4_˙^−^ radicals, amplifying the ECL signal of GDYQDs/ZIF-8.

Multifunctional CRAs rely on synergistic effects at heterogeneous interfaces. Different components separately handle electron transfer, active site exposure, and ROS generation. This achieves multi-mechanism coupling amplification beyond simple addition. Their advantages include simultaneous optimization of activity, stability, and biocompatibility, making them suitable for complex biological sample detection. However, their synthetic routes are complex, and precise control of component ratios remains difficult. Additional bottlenecks include interface compatibility and scalable preparation. Moreover, deciphering structure–activity relationships in multi-component systems is significantly more challenging.

## 3. ECL enhancement mechanisms

The signal amplification effect exerted by CRAs in ECL systems originates from three synergistic catalytic pathways, which can function independently or cooperatively to break through the kinetic and thermodynamic constraints of traditional ECL reactions. These targeted catalytic mechanisms lay a solid theoretical foundation for the rational design and performance regulation of high-efficiency CRAs.

### 3.1 Radical intermediate generation mechanism

This pathway represents the most dominant and fundamental catalytic mechanism of CRAs. In conventional ECL systems, co-reactants typically undergo sluggish electrochemical redox reactions on the electrode surface, yielding only a small quantity of short-lived active radicals (*e.g.*, SO_4_˙^−^, ˙OH, O_2_˙^−^, TPrA˙, and aminoalkyl radicals).^61^ These radicals have an extremely high quenching probability in bulk solution, limiting the overall ECL efficiency. As high-efficiency electrocatalysts, CRAs effectively lower the activation energy required for co-reactant bond cleavage, thereby accelerating the generation of high-concentration, stable active radicals.

**Fig. 4:**
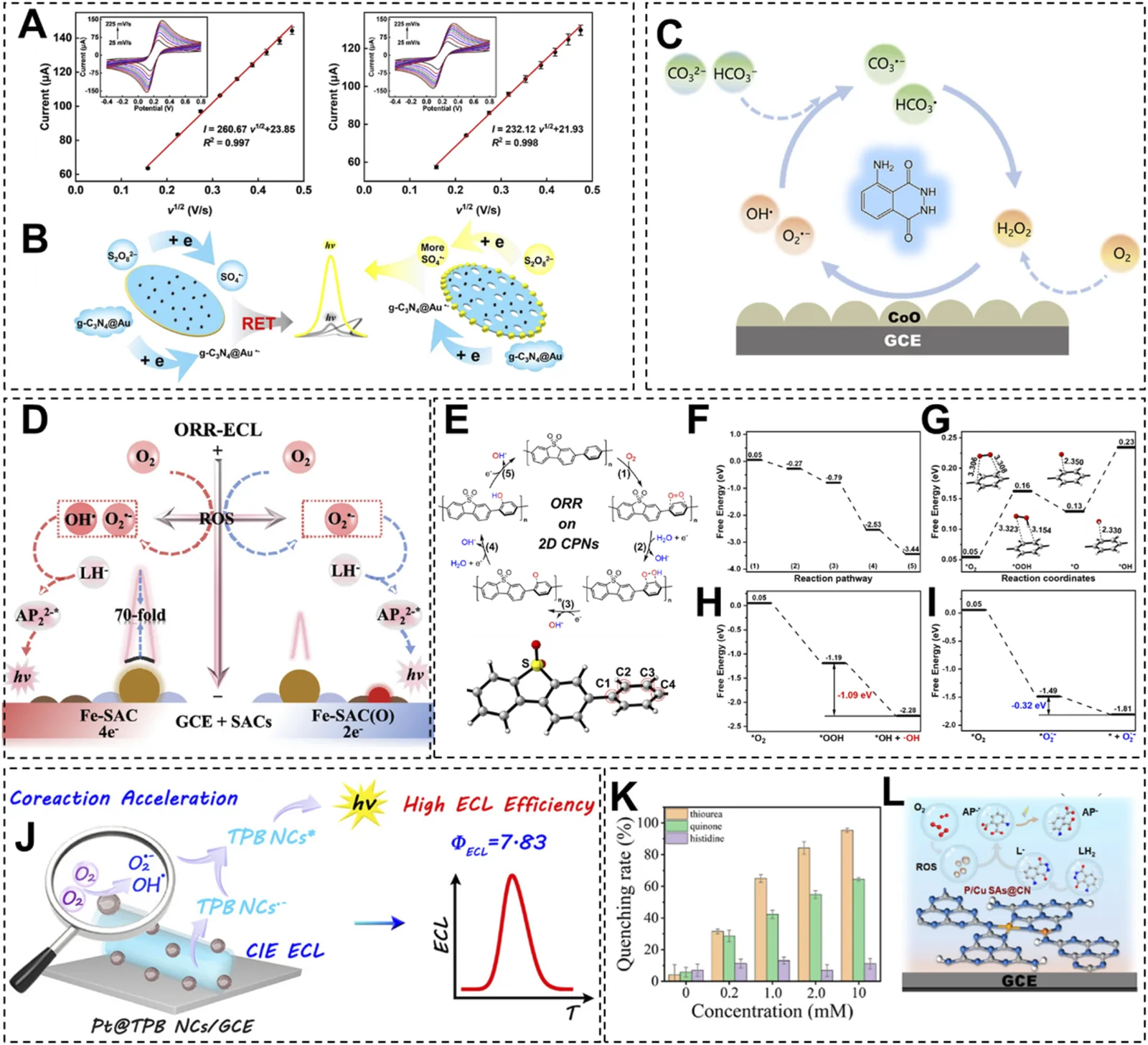
CV curves and linear relations of GCE modified with (A) AuAgPt NFs and (B) AuAgPt NSs and (B) ECL mechanism of the ECL aptasensor. Reproduced with permission from ref. 59. Copyright (2025) American Chemical Society. (C) Schematic diagram of the mechanism of luminol cathodic ECL in carbonate buffer at the CoO NR-modified electrode. Reproduced with permission from ref. 62. Copyright (2023) American Chemical Society. (D) Mechanistic illustration of the luminol–dissolved O_2_ cathodic ECL system with Fe–SAC. Reproduced with permission from ref. 63. Copyright (2022) American Chemical Society. (E) Schematic illustration of 2D CPNs catalyzed ORR process; (F) free energy of the ORR pathway catalyzed by 2D CPNs; (G) adsorption energy of adsorbed intermediates (*i.e.*, *O_2_, *OOH, *O, and *OH); and free energy profiles of: (H) ˙OH and (I) O_2_˙^−^ production pathway for 2D CPNs. Reproduced with permission from ref. 68. Copyright (2026) Wiley-VCH. (J) Mechanistic illustration of Pt@TPB NCs-dissolved O_2_ ECL system. Reproduced with permission from ref. 69. Copyright (2022) American Chemical Society. (K) Quenched signal of the P/Cu SAs@CN luminol–DO system after the addition of various scavengers, including BQ, thiourea, and histidine; and (L) illustration of the Cu single-atom catalysts-involved cathodic ECL mechanism. Reproduced with permission from ref. 70. Copyright (2025) American Chemical Society.

Taking the classic S_2_O_8_^2−^ ECL system as a paradigm, the Cai group proposed a multifunctional ECL co-reaction accelerator—AuAgPt nanoframeworks (NFs).^62^ By modulating the amount of H_2_O_2_, AuAgPt nanosheets (NSs) were etched into AuAgPt NFs, a process in which H_2_O_2_ oxidizes Ag to Ag^+^, disrupting energy resonance transfer. The resulting AuAgPt NFs exhibit a larger effective electroactive surface area than AuAgPt NSs (Fig. 4A) and serve as potent co-reaction promoters. They facilitate the generation of a higher density of SO_4_˙^−^, ultimately enhancing the ECL response of the g-C_3_N_4_@Au/K_2_S_2_O_8_ system (Fig. 4B).

For luminol ECL systems, the low reaction efficiency between luminol and reactive oxygen species (ROS) has historically limited the development of cathodic ECL.^49,63,64^ While state-of-the-art work has focused on improving oxygen reduction reaction (ORR) activity, significant challenges remain. To address this, Xu *et al.* established a synergistic signal amplification pathway for luminol cathodic ECL.^65^ This synergy relies on H_2_O_2_ decomposition *via* catalase-like (CAT-like) CoO nanorods (CoO NRs) and H_2_O_2_ regeneration through a carbonate/bicarbonate buffer using Fe_2_O_3_ nanorods (Fe_2_O_3_ NRs) and NiO microsphere-modified glassy carbon electrodes (GCEs). Compared with the Fe_2_O_3_ NR/NiO-modified GCE, the ECL intensity of the luminol–O_2_ system on the CoO NR-modified GCE was enhanced by nearly 50-fold within the potential range of 0 to −0.4 V. Mechanistically, CAT-like CoO NRs decompose the electroreduction product H_2_O_2_ into ˙OH and O_2_˙^−^, which subsequently oxidize HCO_3_^−^ and CO_3_^2−^ to HCO_3_ and CO_3_˙^−^. These radicals efficiently react with luminol to form luminol radicals. Crucially, the dimerization of HCO_3_˙ generates excited-state 
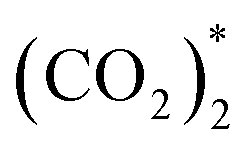
 while regenerating H_2_O_2_, providing a cyclic amplification mechanism for the cathodic ECL signal (Fig. 4C).

Although luminol-based ECL can be excited by various ROS derived from ORR, the presence of multiple reactive intermediates in complex nanomaterials often obscures the specific role of ROS. Moreover, weak cathodic ECL is typically overlooked due to the lack of direct luminol oxidation at the cathode.^63^ Herein, SACs with their tunable coordination environments, were employed to elucidate the relationship between intrinsic ORR activity and ECL behavior.^66^ Remarkably, the traditionally negligible cathodic ECL of luminol was enhanced for the first time (∼70-fold) by combining SAC-catalyzed electrochemical ORR with the chemical oxidation of luminol. By modulating the coordination environment of the central metal atom to selectively generate specific reactive intermediates, the enhanced cathodic ECL exhibited a clear electron transfer pathway-dependent response (Fig. 4D).

The application of 2D conjugated polymers (2D CPs) in ECL has been hindered by inherent defects, such as a lack of catalytic active sites and low electron/hole (e^−^/h^+^) recombination efficiency.^67,68^ In particular, 2D CPs usually exhibit poor ORR performance, limiting ROS generation.^69,70^ To overcome this, the Cai group designed a series of novel 2D CP nanosheets with excellent oxygen evolution activity to serve as efficient co-reaction accelerators.^71^ These nanosheets provided effective catalytic sites that facilitated ORR, boosting the cathodic ECL in the luminol–dissolved O_2_ system with a signal amplification of nearly 50-fold. Notably, within a narrow cathodic potential range (0 to −0.4 V), the four-electron (4e^−^) pathway dominated the ORR process (Fig. 4E–G). DFT calculations further confirmed that the generated ROS (˙OH and O_2_˙^−^) participate in the chemical oxidation of luminol (Fig. 4H and I).

Beyond traditional accelerators, novel luminophores with intrinsic catalytic activity have also been developed. Liu *et al.* synthesized Pt@tetraphenyl-1,3-butadiene nanocrystals (Pt@TPB NCs) which possess high carrier density and electron mobility for efficient e^−^/h^+^ recombination.^72^ Simultaneously, they act as co-reaction promoters for endogenous dissolved O_2_, generating abundant ROS that further interact with TPB NCs. Impressively, the Pt@TPB NCs utilizing dissolved O_2_ as a co-reactant exhibited a *Φ*_ECL_ 7.83 times higher than that of the Ru(bpy)_3_^2+^/dissolved O_2_ system (Fig. 4J).

Furthermore, through coordinated microenvironment modulation, heteroatom P was doped into a Cu single-atom catalyst (P/Cu SAs@CN) to directly trigger cathodic ECL.^73^ The P/Cu SAs@CN catalyst promoted a three-electron ORR pathway, generating abundant ROS, particularly ˙OH, which amplified the ECL emission *via* the oxidation of luminol anions (Fig. 4K and L).

Collectively, these studies—verified through *in situ* electron paramagnetic resonance (EPR), electrochemical characterization, and DFT calculations—have established a quantitative correlation among radical concentration, radical lifetime, and ECL intensity amplification. This confirms that the radical intermediate generation mechanism is the key factor governing the signal amplification efficiency of CRAs.

### 3.2 Interfacial electron transfer mechanism

In addition to accelerating radical generation, CRAs enhance ECL by optimizing interfacial charge transfer kinetics. High-performance CRAs generally exhibit superior electrical conductivity, tunable electronic structures, and abundant surface electroactive sites, which collectively accelerate the heterogeneous electron transfer (HET) rate at the electrode/electrolyte interface.^74,75^ In conventional ECL systems, sluggish electron transfer among the electrode, luminophore, and co-reactant leads to high reaction overpotential and low energy conversion efficiency. CRAs address this by constructing rapid electron transfer channels at the interface, thereby shortening the electron migration distance and minimizing energy loss. In addition, CRAs can suppress the recombination of photogenerated carriers in luminophores (*e.g.*, semiconductor quantum dots and conjugated polymers) and eliminate non-radiative quenching sites, ensuring that electrochemical energy is preferentially converted into luminescent energy rather than heat.

**Fig. 5:**
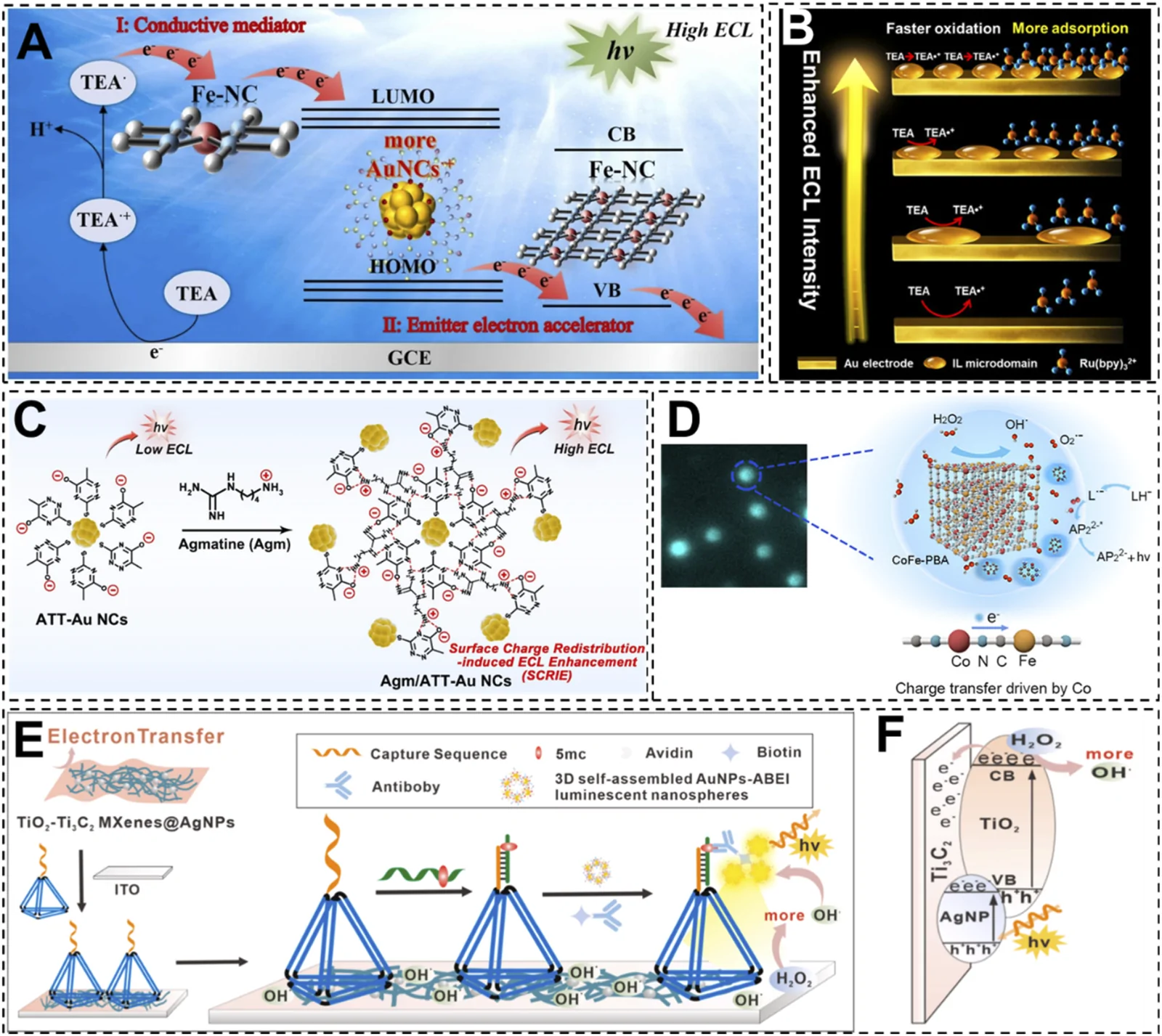
(A) Dual-functional Fe–NC single-atom catalyst as conductive mediator and emitter electron accelerator to enhance the electrochemiluminescence of gold nanoclusters–triethylamine system. Reproduced with permission from ref. 73. Copyright (2026) Elsevier. (B) Schematic illustration of IL microdomains for ECL signal amplification. Reproduced with permission from ref. 75. Copyright (2026) American Chemical Society. (C) Schematic of the preparation and ECL enhancement mechanism of Agm/ATT-Au NCs. Reproduced with permission from ref. 76. Copyright (2025) American Chemical Society. (D) Schematic illustration of CoFe-PBA-catalyzed H_2_O_2_ decomposition into ROS, enhancing the ECL emission of luminol. Reproduced with permission from ref. 77. Copyright (2026) American Chemical Society. (E) Principle of the dual-enhanced electron transfer ECL biosensor; and (F) principle of electron transfer enhanced mechanism of substrate of the ECL biosensor. Reproduced with permission from ref. 78. Copyright (2026) Elsevier.

Liu *et al.* designed a bifunctional iron–nitrogen–carbon single-atom catalyst (Fe–NC SAC) that enhances the ECL of Au nanoclusters (AuNCs) by serving simultaneously as a conductive medium and an emission electron accelerator.^76^ As a conductive medium, Fe–NC substantially reduces the interfacial electron transfer resistance, facilitating electron injection from triethylamine (TEA) into the LUMO level of AuNCs. As an emission electron accelerator, the energy levels of Fe–NC are well aligned with those of AuNCs, enabling rapid extraction of electrons from the HOMO level of AuNCs to the electrode *via* the valence band of Fe–NC. These two effects synergistically accelerate the formation of AuNCs radicals, yielding a 2.23-fold enhancement in ECL intensity (Fig. 5A).

The efficiency of ECL is often limited by insufficient electron transfer at the electrode surface and inadequate reactant accumulation.^77^ To address this, Li *et al.* constructed tunable ionic liquid (IL)-based microdomains on a gold electrode to enhance the ECL performance of the Ru(bpy)_3_^2+^/TEA system in neutral solution.^78^ By varying the alkyl chain length of the IL, they achieved systematic control over the interfacial microdomain size, which produced markedly different ECL enhancements: the smallest microdomain yielded a 20-fold enhancement relative to the bare electrode, whereas the largest microdomain exhibited only a 6-fold enhancement. Electrochemical and ECL results indicate that the IL microdomains accelerate TEA oxidation kinetics and increase the interfacial enrichment of Ru(bpy)_3_^2+^ (Fig. 5B).

Zhu *et al.* developed guanidinobutylamine (Agm)/6-aza-2-thiopyrimidine (ATT)-templated Au NCs (Agm/ATT-Au NCs) as a novel ECL emitter operating *via* the annihilation pathway.^79^ Notably, the positively charged Agm triggers surface charge redistribution on ATT-Au NCs, enhancing the electron transfer kinetics of the emitter and thereby strengthening its electrochemical redox reactions. As a result, the ECL signal of Agm/ATT-Au NCs is approximately 110 times higher than that of ATT-Au NCs, fully demonstrating the excellent co-reactant promotion effect of Agm (Fig. 5C).

The coordination environment of metal centers is crucial for the catalytic performance of catalysts such as Prussian blue analogs (PBAs). However, elucidating their structure–activity relationships at the single-particle level under practical working conditions remains a significant challenge. Gao *et al.* combined electrochemiluminescence microscopy (ECLM) with density functional theory (DFT) calculations to systematically investigate the catalytic activity of CoFe-PBA at the single-particle level for the first time.^80^ DFT calculations revealed that cobalt doping effectively reduces the energy gap (Δ*E*) between occupied N 2p orbitals and unoccupied metal 3d orbitals, facilitating electron transfer from cobalt to iron *via* cyanide bridges. Under electrochemical bias, this doped configuration enables the cobalt center to accelerate interfacial electron transfer and induce metal ion valence state transitions. The resulting rapid charge transfer activates an efficient Fenton-like reaction at cobalt sites, catalyzing the continuous generation of ROS from H_2_O_2_; these radicals subsequently trigger luminol to produce intense ECL signals (Fig. 5D).

Tian *et al.* employed DNA tetrahedron nanostructure probes as CRAs to overcome the poor adaptability caused by low binding efficiency and large steric hindrance in complex clinical samples.^81^ They used a TiO_2_–Ti_3_C_2_ MXene@AgNPs hybrid nanocomposite, rich in active sites, as the electroactive substrate. Accelerated electron transfer in this nanocomposite is attributed to the inherent photoelectric activity of TiO_2_ and the surface plasmon resonance (SPR) effect between AgNPs and TiO_2_, which collectively shorten the reaction pathway. Additionally, three-dimensional (3D) self-assembled AuNPs–ABEI nanoluminophores significantly enhance luminescence intensity by enriching more AuNPs to accelerate electron transfer while simultaneously immobilizing more ABEI molecules (Fig. 5E and F).

In summary, CRAs enhance ECL through diverse interfacial electron transfer strategies. These include conductive mediation, microdomain engineering, surface charge redistribution, and single-particle-level catalytic activation. Although the mechanisms differ, all of these strategies converge on two goals: acceleration of charge migration and suppression of energy loss at the electrode/electrolyte interface. This mechanism complements the radical intermediate generation pathway (Section 3.1), collectively accounting for the majority of signal amplification achieved by CRAs in ECL systems.

### 3.3 Adsorption and confinement mechanism

Most nanomaterial-based CRAs possess ultra-high specific surface area, controllable porous structures, and abundant surface functional groups, enabling efficient adsorption and enrichment of luminophore and co-reactant molecules on the material surface.^82,83^ This creates a locally high-concentration reaction microenvironment around the CRA, which shortens the diffusion distance of active radicals and reduces quenching losses caused by long-distance migration in bulk solution. Concurrently, the spatial confinement effect restricts the free movement of short-lived active radicals, prolongs their effective lifetime, and increases the probability of productive collisions between radicals and luminophores.^84,85^

**Fig. 6:**
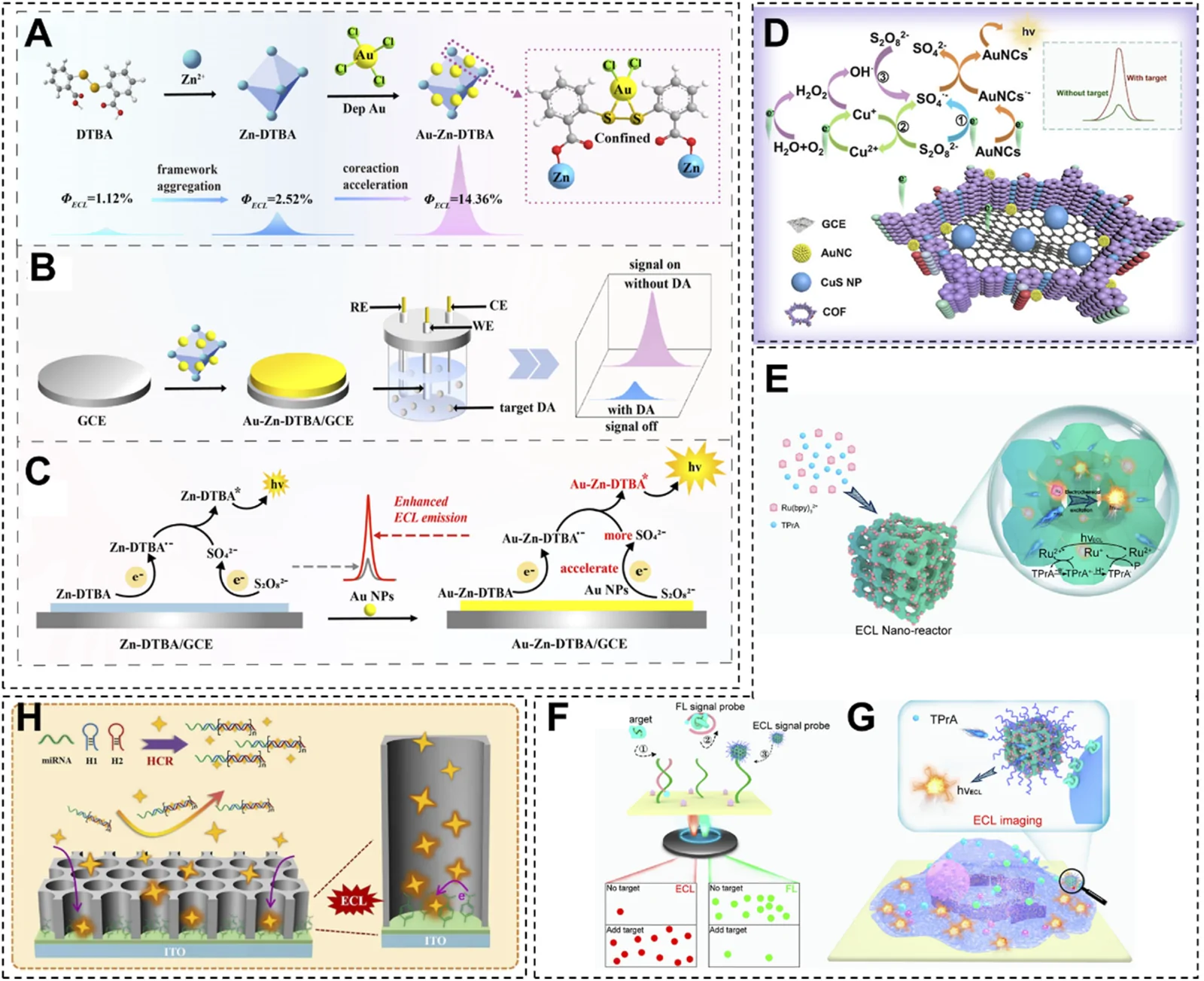
(A) Schematic synthesis of the Au–Zn–DTBA and corresponding ECL efficiency of pure 2,2′-dithiodibenzoic acid (DTBA), framework-enhanced DTBA, and confined DTBA; (B) fabrication of the AuNPs-accelerated ECL biosensor for detection of dopamine (DA); and (C) comparison of the ECL mechanism in the absence and presence of a CRA. Reproduced with permission from ref. 81. Copyright (2025) American Chemical Society. (D) Schematic of ECL amplification mechanism based on active radicals enriched by nanoconfinement effect synergistically with CRA. Reproduced with permission from ref. 82. Copyright (2025) American Chemical Society. (E) Schematic mechanism for the nanoconfinement-enhanced ECL reaction at single nanoreactors; (F) dual-signal imaging platform for imaging tracking of individual target molecules; and (G) ECL imaging of single target proteins on the cell membrane. Reproduced with permission from ref. 83. Copyright (2025) Elsevier. (H) Schematic illustration for homogeneous ECL bioassay based on VMSF/N-ITO sensing platform. Reproduced with permission from ref. 84. Copyright (2023) Elsevier.

Song *et al.* synthesized an efficient ECL coordination polymer, Au–Zn–DTBA (Fig. 6A), using 2,2′-dithiodibenzoic acid (DTBA) as the luminescent ligand, Zn^2+^ as the metal center, and Au nanoparticles (AuNPs) as CRAs anchored onto DTBA *via* Au–S bonds.^86^ Based on this coordination polymer, they constructed a novel biosensor for the sensitive detection of dopamine, a biomarker of neurological diseases (Fig. 6B). Compared with the Zn–DTBA system without CRAs, the ECL intensity of Au–Zn–DTBA was enhanced by 4-fold. This enhancement arises because the AuNPs confined within the Zn–DTBA framework facilitate the activation of K_2_S_2_O_8_ to generate abundant sulfate radicals (SO_4_˙^−^), thereby significantly improving the ECL efficiency of Zn–DTBA (Fig. 6C).

Low electron transfer efficiency and energy loss during mass transport are fundamental bottlenecks limiting ECL performance. To address this, Xu *et al.* proposed a synergistic strategy that combines nanoconfinement with co-reactant acceleration.^87^ Specifically, copper sulfide nanoparticles (CuS NPs) were encapsulated within a covalent organic framework (COF) *via* an embedding method, followed by *in situ* synthesis of AuNCs within the COF pores, yielding the high-performance ECL emitter AuNCs@CuS@COF. In this system, CuS NPs serve as efficient CRAs, catalyzing persulfate to generate abundant reactive radicals, while the nanoconfinement effect of the COF enhances the interaction between these radicals and the emissive AuNCs, resulting in a substantial boost in ECL emission intensity (Fig. 6D).

Beyond signal amplification, the confinement mechanism can also be directly visualized at the single-particle level. Lu *et al.* developed nanoreactors based on Ru(bpy)_3_^2+^-doped nanoporous zeolite nanoparticles (Ru@zeolite) for this purpose.^88^ Each nanoreactor serves simultaneously as a host matrix for Ru(bpy)_3_^2+^-ions, a nanoconfined reaction environment, and a source of co-reactant radicals, enabling efficient *in situ* ECL generation (Fig. 6E). The nanoconfinement effect significantly enhances the ECL signal. Using these nanoreactors as ECL probes, the authors established a dual-signal sensing protocol for the visual tracking of individual biomolecules (Fig. 6F and G). Xu *et al.* first proposed an innovative strategy for regulating ECL using silicon nanochannels. *N*,*N*-Dimethylaniline (DMA) was electrografted onto the ITO electrode surface and acted as the underlying coreactant layer. Also, a vertically ordered mesoporous silica film (VMSF) was fabricated to serve as the efficient CRAs for modulating the ECL emission of Ru(bpy)_3_^2+^ (Fig. 6H).^89^

Collectively, these studies demonstrate that the adsorption and confinement mechanism enhances ECL by creating a locally concentrated reaction microenvironment that shortens radical diffusion pathways and prolongs radical lifetimes. This mechanism integrates coordination polymer-based enrichment, COF-enabled nanoconfinement, and single-particle visualization. It complements the radical generation and interfacial electron transfer pathways, offering a versatile strategy for high-performance ECL sensing.

To sum up, these three mechanisms, including radical intermediate generation, interfacial electron transfer, and adsorption-confinement, are not mutually exclusive but rather operate synergistically in most practical ECL systems. However, their relative contributions and applicability vary significantly depending on the reaction system and detection scenario.

The radical intermediate generation mechanism (Section 3.1) is the most universally applicable and dominant pathway, as it directly addresses the fundamental bottleneck of low co-reactant conversion efficiency. Nevertheless, its effectiveness is intrinsically limited by the catalytic activity of the CRA and the short lifetime of radical intermediates, which constrain the spatial range of signal amplification. The interfacial electron transfer mechanism (Section 3.2) is particularly advantageous in systems where sluggish charge transfer at the electrode interface is the rate-limiting step, such as in luminol-based cathodic ECL and quantum dot-based anodic ECL. However, this mechanism is highly dependent on the intimate contact between the CRA and the electrode surface, and its contribution diminishes when the CRA is spatially separated from the luminophore. The adsorption-confinement mechanism (Section 3.3) offers a unique advantage in creating locally concentrated reaction microenvironments, making it especially effective for single-particle-level ECL amplification and bioimaging applications. Yet, its generalizability is limited by the specific pore architecture and surface chemistry of the CRA, and it is less effective in homogeneous solution-phase systems. In practice, the most effective CRAs, such as MOF-based composites and COF-confined nanostructures, leverage two or even all three mechanisms simultaneously, suggesting that future CRA design should prioritize multi-mechanism integration rather than optimization of a single pathway.

## 4. Biosensing applications for advanced CRAs

Benefiting from exceptional signal amplification efficiency, advanced CRAs have been widely applied in various high-performance ECL bioanalytical platforms, covering major categories of biological targets and meeting the needs of clinical diagnosis, environmental monitoring, and food safety (Table 1). The following sections summarize typical application scenarios and core performance indicators.

Biosensing applications with advanced CRAs.

**Table:** 

CRAs	Mechanism	Disease biomarker	Detection range	LOD	Ref.
ZIF-67@MXene	Radical intermediate generation and adsorption mechanism	Prostate-specific antigen (PSA)	1.44 × 10^−11^ to 9.1 × 10^−13^ g mL^−1^	9.1 × 10^−13^ g mL^−1^	47
CeO_2_–Pt	Radical intermediate generation mechanism	CA15-3	5 × 10^−4^ to 50 U mL^−1^	5.75 × 10^−5^ U mL^−1^	91
α-Fe_2_O_3_–Pt	Radical intermediate generation mechanism	Procalcitonin (PCT)	10 fg mL^−1^ to 100 ng mL^−1^	3.56 fg mL^−1^	92
FN-GQDs	Radical intermediate generation mechanism	Tumor necrosis factor-like cytokine 1A (TL1A)	0.50 to 100.00 ng mL^−1^	0.33 ng mL^−1^	94
Ru-MOF-5	Radical intermediate generation mechanism	Neuron-specific enolase (NSE)	0.0001 ng mL^−1^ to 200 ng mL^−1^	0.041 pg mL^−1^	95
CoSn(OH)_6_-Au	Interfacial electron transfer mechanism	Serum amyloid A (SAA)	10 fg‧mL^−1^ to 100 ng‧mL^−1^	2.6 fg‧mL^-1^	96

**Table:** 

CRAs	Mechanism	Environmental pollutant	Detection range	LOD	Ref.
Nd-MOFs	Radical intermediate generation mechanism	Pb^2+^	1 µM to 10 fM	4.1 fM	97
Ag_3_PO_4_@PTCA	Radical intermediate generation mechanism	Pb^2+^	1 µM to 10 fM	0.253 fM	98
2-(Dibutylamino)ethanol (DBAE)	Confinement mechanism	Microcystin-LR (MC-LR)	50 fg mL^−1^ to 10 ng mL^−1^	31 fg mL^−1^	99
IRMOF-3	Confinement and radical intermediate generation mechanism	Profenofos (Pff)	134 fM to 1.34 mM	44.7 fM	100

**Table:** 

CRAs	Mechanism	Food safety hazards	Detection range	LOD	Ref.
ZCM@SnS_2_	Interfacial electron transfer and radical intermediate generation mechanism	Fumonisin B1 (FB1)	10 pg mL^−1^ to 100 ng mL^−1^	3.9 pg mL^−1^	101
CoZn-ZIF@MWCNTs	Radical intermediate generation mechanism	Ochratoxin A (OTA)	0.1 to 10^4^ nM	0.023 nM	102
Ag AGs	Radical intermediate generation mechanism	Aflatoxin B1 (AFB1)	1 pg mL^−1^ to 100 ng mL^−1^	0.23 pg mL^−1^	103
RI-MOF	Interfacial electron transfer mechanism	AFB1	0.01 ng mL^−1^ to 100 ng mL^−1^	3.35 pg mL^−1^	104

### 4.1 Disease biomarker detection

The ultrasensitive detection of disease biomarkers is a core requirement for clinical early diagnosis.^90^ The introduction of advanced CRAs into ECL biosensing systems has opened new avenues for the ultra-sensitive and rapid detection of trace-level targets, including low-abundance proteins, circulating nucleic acids, and exosomes. Representative applications are discussed below.

**Fig. 7:**
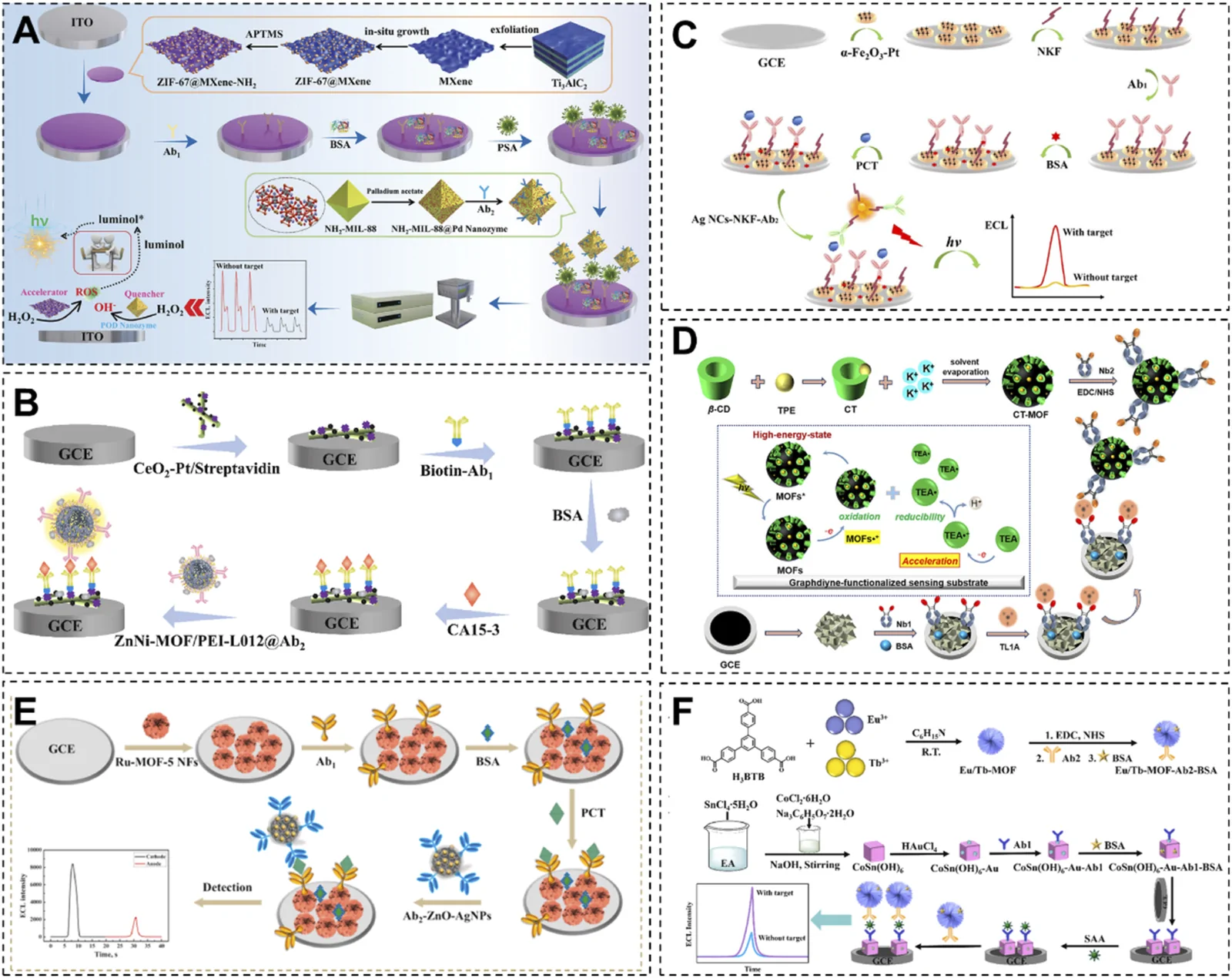
(A) Schematic illustration of the ECL immunoassay assisted by the synergy of highly efficient co-reaction accelerator and nanozyme-based quenching transducer. Reproduced with permission from ref. 43. Copyright (2024) Wiley-VCH. (B) Schematic diagram of CeO_2_–Pt as CRA for CA15-3 detection. Reproduced with permission from ref. 86. Copyright (2025) Elsevier. (C) Schematic diagram of the biosensor construction process. Reproduced with permission from ref. 87. Copyright (2023) American Chemical Society. (D) Synthesis route of the CT-MOF and schematic illustration of the TL1A nanobody-based immunosensor, including the ECL sensing mechanism. Reproduced with permission from ref. 89. Copyright (2026) Wiley-VCH. (E) Schematic diagram of ECL immunosensor fabrication for neuron-specific enolase detection. Reproduced with permission from ref. 90. Copyright (2021) Elsevier. (F) Synthesis of Eu/Tb-MOF-Ab2 and preparation of CoSn(OH)_6_–Au-Ab1 and ECL immunosensor for detecting SAA. Reproduced with permission from ref. 91. Copyright (2026) Elsevier.

Wang *et al.* designed ZIF-67@MXene nanosheet composites, featuring highly conductive in-plane structures and confinement-stabilized pores/channels, as efficient CRAs in a luminol–H_2_O_2_-based ECL system.^47^ Leveraging this system, they further constructed a “switch-on” immunosensor for prostate-specific antigen (PSA) using NH_2_-MIL-88(Fe)@Pd plasmonic nanozymes (Fig. 7A). Similarly, Cui *et al.* synthesized polyethylenimine (PEI)-grafted bimetallic ZnNi-MOF nanospheres (ZnNi-MOF/PEI) and constructed an L012-based ECL immunosensor for carbohydrate antigen 15-3 (CA15-3) by using Pt nanoparticle-functionalized CeO_2_ nanorods (CeO_2_–Pt) as efficient CRAs (Fig. 7B).^91^ Song *et al.* developed a highly efficient biosensor based on a ternary ECL system with α-Fe_2_O_3_–Pt as advanced CRAs for procalcitonin (PCT) detection (Fig. 7C).^92^ Tetraphenylethylene (TPE) exhibits aggregation-induced ECL (AIECL), but achieving high emission efficiency requires effective suppression of non-radiative decay.^93^ Du *et al.* addressed this by constructing a cyclodextrin–TPE metal–organic framework (CT-MOF) that enhances ECL *via* a coordination-induced ECL (CIECL) mechanism.^94^ Notably, they developed a nanobody-based sandwich immunosensor for the sensitive detection of tumor necrosis factor-like cytokine 1A (TL1A, a key biomarker for colitis) by the introduction of FeNi alloy-decorated graphdiyne quantum dots (FN-GQDs) as CRAs (Fig. 7D). Dong *et al.* designed a dual-signal ratiometric ECL sensing strategy for neuron-specific enolase (NSE) with Ru-MOF-5 as CRAs (Fig. 7E).^95^ Compared with traditional biomarkers such as C-reactive protein (CRP), serum amyloid A (SAA) serves as a more sensitive indicator for early infection detection. Zheng *et al.* developed a highly sensitive ECL immunosensor based on a lanthanide bimetallic MOF (Eu/Tb-MOF) integrated with a CoSn(OH)_6_–Au nanocomposite (serving as a CRA) for SAA quantification (Fig. 7F).^96^

Collectively, these studies demonstrate that CRAs significantly expand the analytical performance of ECL biosensors. This is achieved by enabling diverse signal amplification strategies, including sandwich-type, ternary, ratiometric, and nanobody-based ECL platforms. As a result, ultralow detection limits have been attained for a broad range of clinical biomarkers.

### 4.2 Environmental pollutant detection

Environmental toxins include heavy metal ions, pesticide residues, and persistent organic pollutants. Detecting them in complex environmental samples is challenging due to strong background interference. Advanced CRAs address this issue with their remarkable signal amplification capability. They effectively suppress background noise and significantly improve the sensitivity and reliability of environmental biosensors. These biosensors are well suited for on-site and rapid detection. Representative applications are discussed below.

**Fig. 8:**
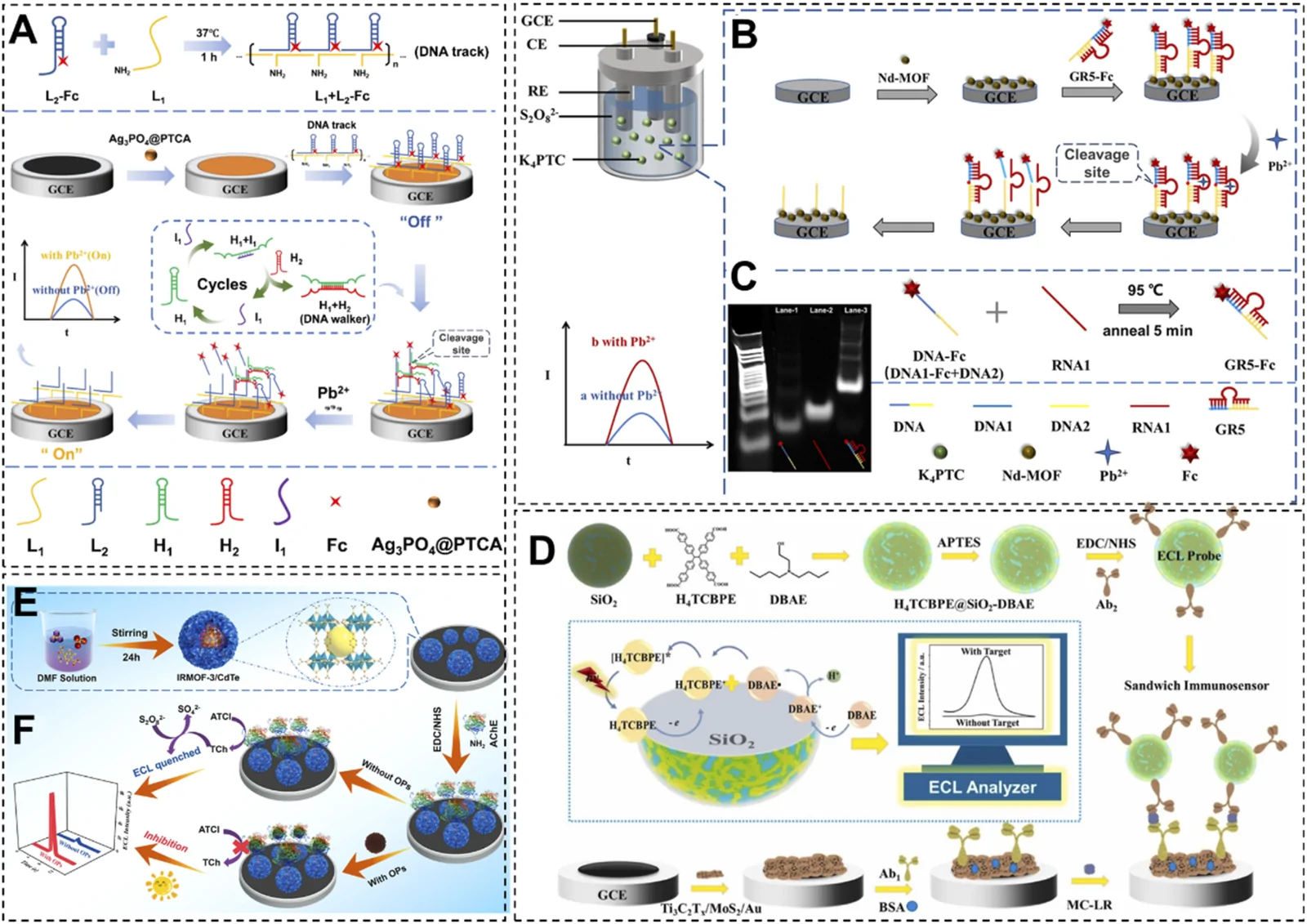
(A) Preparation of the DNA tracks in the DNA walker and Pb^2+^ detection process of the ECL biosensor. Reproduced with permission from ref. 92. Copyright (2024) American Chemical Society. (B) Pb^2+^ detection process of the ECL biosensor; and (C) gel electrophoresis image of DNA-Fc (DNA1-Fc + DNA2), RNA1, and GR5-Fc. Reproduced with permission from ref. 93. Copyright (2025) Elsevier. (D) Schematic diagram of the construction of H_4_TCBPE@SiO_2_–DBAE as a self-enhanced ECL probe for MC-LR assay. Reproduced with permission from ref. 94. Copyright (2024) Elsevier. (E) Synthesis process of IRMOF-3/CdTe and (F) manufacturing process of the ECL enzyme biosensor. Reproduced with permission from ref. 95. Copyright (2025) Elsevier.

A ternary ECL biosensor was developed based on DNAzyme for the ultrasensitive detection of Pb^2+^ with neodymium-based metal–organic frameworks (Nd-MOFs) as novel CRA (Fig. 8A).^97^ Building on this strategy, our group further designed a novel ECL biosensor based on a track-driven DNA walker for Pb^2+^ detection using Ag_3_PO_4_@perylene tetracarboxylic acid (PTCA) as efficient CRA (Fig. 8B and C).^98^ Similarly, Du *et al.* developed an ECL immunosensor for microcystin-LR (MC-LR), an algal toxin with multi-organ toxicity, genotoxicity, and carcinogenicity, based on an aggregation-induced ECL (AIECL)-assisted self-enhancement strategy with 2-(dibutylamino)ethanol (DBAE) as CRAs (Fig. 8D).^99^ Leveraging the superior ECL efficiency of IRMOF-3/CdTe, together with the inhibitory effect of organophosphorus pesticides (OPs) on acetylcholinesterase (AChE) activity, a highly sensitive ECL enzymatic biosensor was constructed with IRMOF-3 serving as efficient CRA for the detection of profenofos (Pff) (Fig. 8E and F).^100^

Collectively, these studies demonstrate that CRAs enable ultrasensitive detection of both heavy metal ions and organic pollutants in complex environmental matrices, with detection limits reaching the femtomolar to femtogram-per-milliliter level, underscoring their practical value for on-site environmental monitoring.

### 4.3 Food safety monitoring

For food safety monitoring, ECL biosensing platforms based on advanced CRAs have been successfully applied to the rapid screening of various harmful species, including antibiotics, mycotoxins, foodborne pathogenic bacteria, and illegal additives. These platforms achieve an effective balance between high-sensitivity signal output and the convenience of on-site operation. Representative applications are discussed below.

**Fig. 9:**
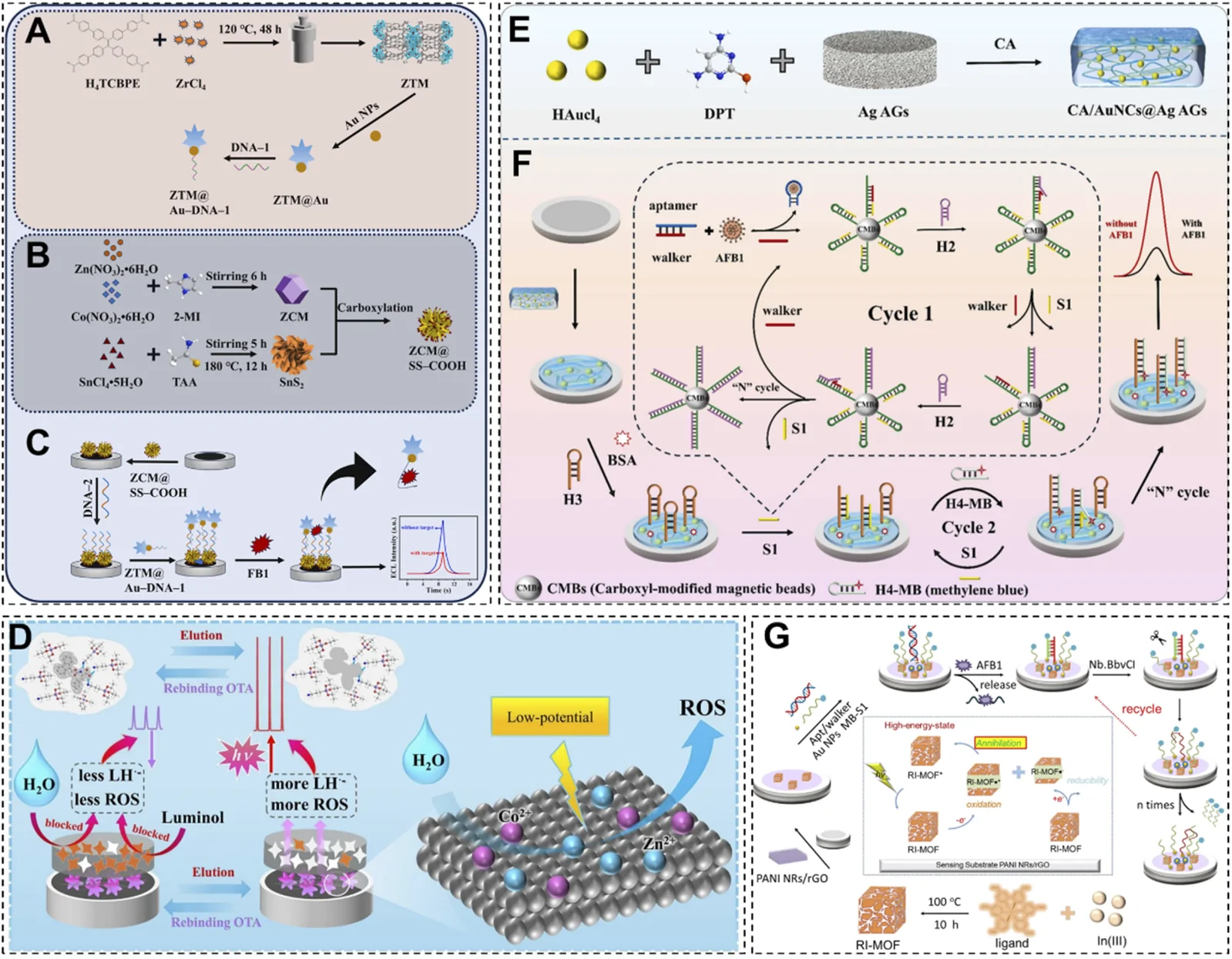
(A) Synthesis of ZTM@Au–DNA-1 bioconjugates; (B) synthesis of ZCM@SnS_2_–COOH and (C) fabrication process of the ECL aptasensor. Reproduced with permission from ref. 96. Copyright (2026) Elsevier. (D) Schematic illustration of the proposed molecularly imprinted electrochemiluminescence sensor for ochratoxin A (OTA) detection. Reproduced with permission from ref. 97. Copyright (2026) Elsevier. (E) Synthesis approach of CA/AuNCs@Ag AGs; and (F) construction of biosensor for enzyme-free catalytic double-cycle amplification strategy. Reproduced with permission from ref. 98. Copyright (2026) Elsevier. (G) Schematic diagram of the ECL biosensor for AFB1 detection based on the Nb.BbvCI endonuclease-driven DNA walker. Reproduced with permission from ref. 99. Copyright (2025) Elsevier.

Accurate detection of fumonisin B1 (FB1) is of great significance for food safety and disease prevention. ZnCo-MOF@SnS_2_ (ZCM@SnS_2_) served as the CRA to construct a dual-MOF ECL sensor that synergistically regulates interfacial electron transfer, enabling ultrasensitive FB1 detection (Fig. 9A–C).^101^ Similarly, Liao *et al.* proposed a luminol–aqueous system enhancement strategy using a multi-walled carbon nanotube (MWCNT)-functionalized bimetallic CoZn-zeolitic imidazolate framework (CoZn-ZIF) as the CRA for ochratoxin A (OTA) detection (Fig. 9D).^102^ Jia *et al.* employed the synergistic aggregation of citric acid (CA) and silver aerogels (Ag AGs) to assemble AuNCs onto Ag AGs, yielding an integrated catalytic-luminescent ECL emitter, CA/AuNCs@Ag AGs (Fig. 9E).^103^ Here, Ag AGs act as CRAs to efficiently link trace aflatoxin B1 (AFB1) with abundant quencher-bearing DNA strands *via* catalytic hairpin assembly, enabling ultrasensitive AFB1 detection through efficient ECL resonance energy transfer (Fig. 9F). Complementing this approach, an independent study developed an annihilation-type ECL system for AFB1 detection, employing a highly symmetric metal–organic framework (RI-MOF) as a novel self-luminescent emitter, as well as CRA and an endonuclease-driven DNA walker as a signal amplifier (Fig. 9G).^104^

Collectively, these CRAs-enhanced ECL biosensors have been successfully applied to monitor hazardous substance residues in food, and the results underscore the promising potential of CRAs-based ECL platforms for practical food safety monitoring.

### 4.4 *In situ* bioanalysis and ECL bioimaging

With the advancement of *in situ* bioanalysis, CRA-enhanced ECL technology has expanded beyond conventional biosensing to *in vitro* real-time monitoring and ECL bioimaging. High-biocompatibility CRAs enable intracellular ROS and biomarker imaging, as well as dynamic monitoring of cell apoptosis and proliferation. Compared with traditional fluorescence imaging, CRA-based ECL imaging offers superior spatial resolution and lower background, effectively overcoming limitations such as photobleaching and autofluorescence. Furthermore, the integration of CRA-ECL with microfluidic chips enables high-throughput, real-time monitoring of cell secretion and drug response, advancing applications in precision medicine and drug screening. Representative advances are discussed below.

**Fig. 10:**
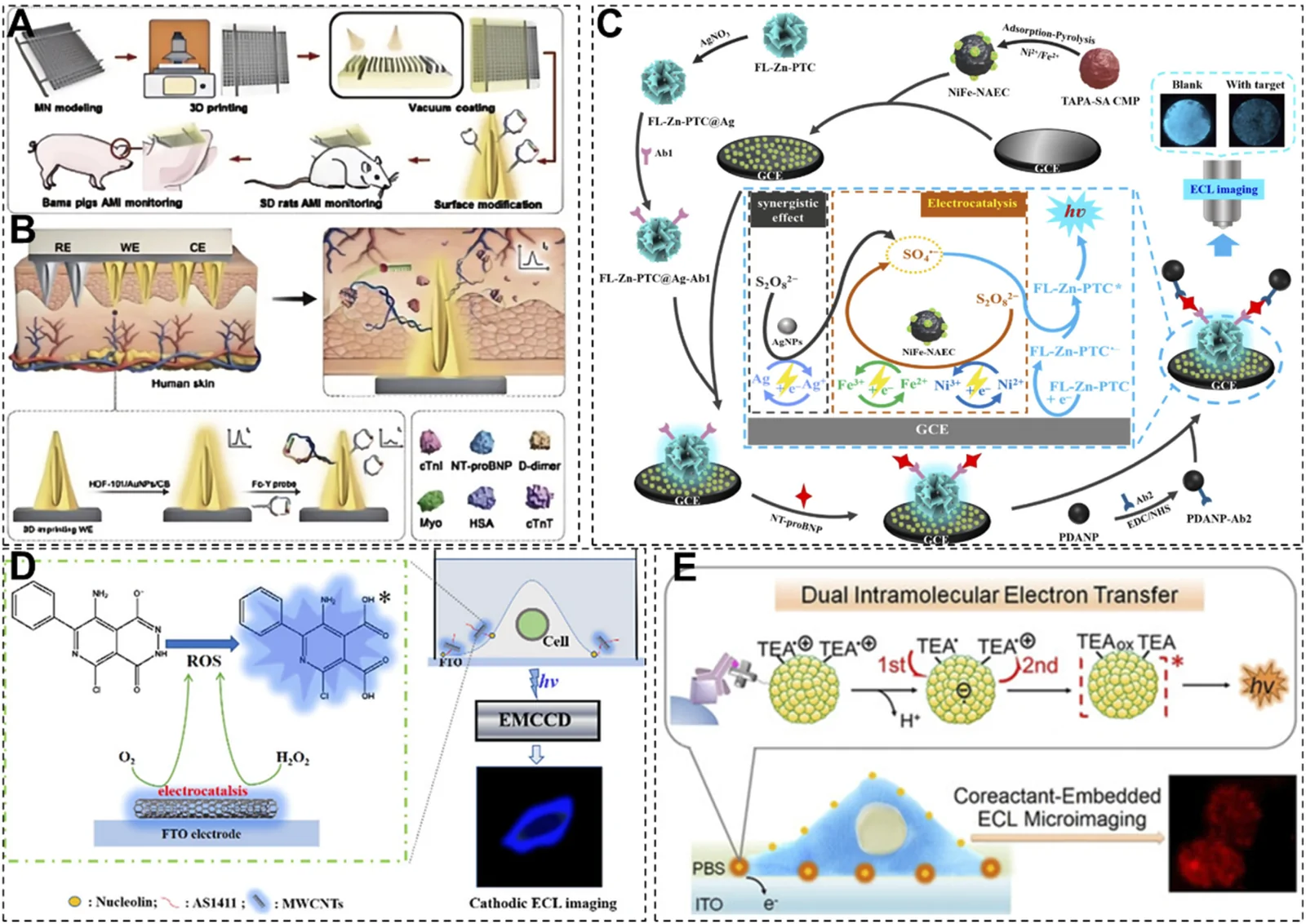
(A) Construction of an ECL MN monitoring system and (B) schematic diagram of the integrated MN sensing. Reproduced with permission from ref. 100. Copyright (2026) Springer Nature. (C) NiFe nanoalloy electrocatalyst as a CRA for enhancing biomarker electrochemiluminescence imaging detection. Reproduced with permission from ref. 101. Copyright (2024) American Chemical Society. (D) Schematic illustration of cathodic ECL microscopy of MWCNTs for ECL microimaging of nucleolin on a single tumor cell. Reproduced with permission from ref. 102. Copyright (2023) American Chemical Society. (E) Schematic illustration of dual intramolecular electron transfer of TEA–Pdots for ECL microimaging of HER2 on single cells. Reproduced with permission from ref. 103. Copyright (2021) Wiley-VCH.

Continuous *in vivo* monitoring of biomarkers remains challenging due to limited sensitivity, integrability, and biocompatibility. Xiong *et al.* addressed this by developing an integrated microneedle electrochemiluminescence device (MN-ECLD) for real-time detection of protein biomarkers in interstitial fluid.^105^ The device incorporates a hydrogen-bonded organic framework (HOF)-loaded ultra-bright, biocompatible ECL emitter into a porous gold-plated microneedle array, regulated by interface-specific Y-shaped probes to enable efficient co-reactant-free ECL signal generation. *In vitro* tests demonstrate ultrasensitive protein detection with a linear range of 100 fg mL^−1^ to 10 ng mL^−1^, a detection limit of 21.3 fg mL^−1^, and stability exceeding 12 days—achieving an 87-fold sensitivity enhancement over conventional emitters. *In vivo*, the MN-ECLD enabled real-time monitoring of cardiac biomarkers, facilitating early warning of acute myocardial infarction in both rats and pigs, with biomarker trends consistent with serum ELISA results. This work establishes a multifunctional platform for continuous *in vivo* diagnostics of acute cardiovascular and metabolic diseases (Fig. 10A and B).

Wu *et al.* employed NiFe nanoalloy electrocatalysts (NiFe-NAEC) as CRAs to activate persulfate, increasing the ECL intensity of flower-like Zn-3,4,9,10-perylenetetracarboxylate (FL-Zn-PTC) by 44-fold.^106^ The synergistic combination of NiFe-NAEC with silver nanoparticles further boosted the ECL intensity by 134-fold, corresponding to a 6275% enhancement in ECL efficiency relative to the Ru(bpy)_3_^2+^/K_2_S_2_O_8_ reference system. Based on this amplified platform, a sensor was constructed for the visual detection of N-terminal pro-B-type natriuretic peptide (NT-proBNP), exhibiting a linear range of 0.01–1000 pg mL^−1^ and a detection limit of 2.1 fg mL^−1^ with intuitive readouts (Fig. 10C).

Beyond bulk detection, CRA-enhanced ECL has been extended to imaging at the single-cell and single-molecule level. Zhang *et al.* developed a cathodic ECL microscope based on the luminol analogue L012 for imaging individual nanotubes and nucleolin on single cells.^107^ The microscope employs multi-walled carbon nanotubes (MWCNTs) as CRAs, which efficiently convert dissolved O_2_ and H_2_O_2_ into ROS owing to their superior electrocatalytic performance. The generated ROS oxidize L012 to its excited state, producing bright cathodic ECL emission at low triggering potentials. After modification with the AS1411 aptamer, the MWCNT@AS1411 probe enabled specific ECL imaging of nucleolin on the plasma membrane of tumor cells, allowing label-free bioassays without ECL tags. This L012-based cathodic ECL microscope, featuring moderate operating potential and label-free capability, provides a universal approach for imaging single nanomaterials and single cells (Fig. 10D).

Wang *et al.* combined a molecular intramolecular electron transfer strategy with tertiary amine-conjugated polymer dots (TEA–Pdots) to develop a co-reactant-embedded ECL mechanism for single-cell micro-imaging.^108^ TEA–Pdots generate ECL emission at +1.2 V without any exogenous co-reactant, and their unique molecular architecture yields unprecedented ECL intensity. Specifically, it reaches 132 times that of an equivalent Pdots/TEA mixture and 45 times that of a Pdots–TEA mixture with 62.5-fold excess TEA. This performance even surpasses that of the conventional Ru(bpy)_3_^2+^ system. This co-reactant-embedded strategy enables *in situ* ECL micro-imaging of membrane proteins on individual living cells without additional permeabilization for co-reactant delivery. The feasibility was validated by evaluating cell-surface-specific protein expression, opening new avenues for ECL applications in single-cell analysis and dynamic studies of biological events (Fig. 10E).

Collectively, these studies demonstrate that CRA-enhanced ECL technology has evolved from bulk biosensing to sophisticated *in situ* bioanalysis platforms. These platforms now encompass continuous *in vivo* monitoring, ultrasensitive disease biomarker detection, and label-free single-cell imaging. Such advances highlight the transformative potential of this technology for both precision medicine and fundamental biological research.

A cross-comparison of the biosensing applications discussed above reveals both the remarkable versatility and the remaining gaps of CRA-enhanced ECL platforms. In terms of analytical performance, disease biomarker detection (Section 4.1) has achieved the lowest detection limits. These limits reach the sub-femtomolar level (*e.g.*, 0.253 fM for Pb^2+^) due to synergistic amplification strategies such as DNA walkers and dual-cycling, which are uniquely suited to nucleic acid and protein targets. Environmental pollutant detection (Section 4.2) and food safety monitoring (Section 4.3) typically operate at higher detection limits (pg mL^−1^ to ng mL^−1^ range), reflecting the greater complexity of real sample matrices and the need for robust selectivity over ultra-high sensitivity. Notably, *in situ* bioimaging and *in vivo* monitoring (Section 4.4) represent the most demanding application scenarios, requiring not only high sensitivity but also excellent biocompatibility, long-term stability, and minimal invasiveness—requirements that remain only partially met by current CRAs.

Several unmet needs persist across all application domains. First, the majority of CRAs reported to date are based on noble metals or complex nanocomposites, raising concerns about cost, scalability, and long-term biotoxicity for *in vivo* applications. Second, while single-target detection has achieved impressive sensitivity, multiplexed detection of multiple biomarkers with high specificity remains scarce, limiting the clinical translatability of these platforms. Third, the integration of CRA-ECL systems with portable, user-friendly devices for true point-of-care testing is still in its infancy, with most demonstrations confined to laboratory settings. Addressing these gaps will be essential for translating CRA-enhanced ECL from a powerful analytical tool into a practical diagnostic technology.

## 5. Conclusion and future perspectives

Advanced CRAs have brought a revolutionary breakthrough to ECL bioanalysis. They overcome the performance bottlenecks of traditional ECL systems and enable ultra-sensitive, rapid, and specific detection of diverse biological targets. This review systematically summarizes the core definition, catalytic mechanisms, material classification, and design strategies of CRAs across inorganic, organic/biomolecular, and multifunctional categories. Their applications span disease diagnosis, food safety, environmental monitoring, and bioimaging.

Despite this progress, several critical challenges remain. First, molecular-level structure–activity relationships of most CRAs are still unclear. Catalytic mechanisms are largely inferred from macroscopic characterization rather than verified by *operando* monitoring of radical intermediates. Second, a persistent cost–stability–activity trade-off constrains material selection. Noble metal CRAs offer high activity but at prohibitive cost. Non-noble alternatives are more economical but suffer from lower stability. Third, biocompatibility concerns limit *in vivo* applications. Most CRA-ECL systems also remain confined to laboratory settings, lacking portable platforms for point-of-care testing.

Addressing these challenges requires coordinated advances across multiple frontiers. AI-assisted CRA design can accelerate the rational discovery of optimal compositions and morphologies. Machine learning models trained on high-throughput synthesis–property datasets can drastically reduce the trial-and-error cycle. Single-atom mechanistic studies must move toward *operando* understanding. This will clarify how isolated metal centers interact with co-reactants at the atomic level under real ECL cycling conditions. Metal-free CRAs, including heteroatom-doped carbons, g-C_3_N_4_, and organic polymer catalysts, represent a promising direction to decouple performance from cost and toxicity. However, their catalytic efficiency still requires systematic optimization.

Looking ahead, the ultimate goal is autonomous, intelligent diagnostics. Integration of CRA-ECL systems with microfluidic chips, paper-based sensors, and wearable devices can enable sample-in-answer-out POCT platforms. Coupling these with smartphone-based readout and on-device AI algorithms will make decentralized diagnosis feasible, especially in resource-limited settings. Multiplexed, wavelength-resolved ECL arrays will be critical for simultaneous multi-biomarker detection. Biodegradable, low-toxicity CRAs, especially biomolecule–inorganic hybrids, are urgently needed for intracellular sensing and short-term *in vivo* bioimaging. Ultimately, standardized synthesis protocols and large-scale clinical validation will be required to translate CRA-ECL technology from benchtop research into practical clinical diagnostics.

## Author contributions

J. X. L. wrote the manuscript. D. Y., R. C., and W. T. designed the structure of the review, and revised the manuscript. All authors have given approval to the final version of the manuscript. We appreciate Dr Kathryn R. Williams's great help in reviewing and correcting the manuscript.

## Conflicts of interest

There are no conflicts to declare.

## Data Availability

No primary research results have been included and no new data were generated or analysed as part of this review.
